# Optimisation and analytical assessment of a TaqMan™ probe-based real-time PCR assay designed to diagnose infection with *Schistosoma japonicum*

**DOI:** 10.1186/s13071-026-07458-2

**Published:** 2026-06-29

**Authors:** John Archer, Sarah Hingel, Charo Abarsosa, Katherine Zabala, Jennifer M. Nailes, Bonnie L. Webster

**Affiliations:** 1 Wolfson Wellcome Biomedical Laboratories, Department of Zoology, Natural History Museum, Cromwell Road, London, UK; 2 Foundation for Innovative and New Diagnostics (FIND), Chemin du Pommier 40, 1218 Grand-Saconnex, Switzerland; 3 Schistosomiasis Research Training Center, Palo, Leyte Philippines; 4 University of the East Ramon Magsaysay Memorial Medical Center, Research Institute for the Health Sciences, Quezon City, Philippines

**Keywords:** Intestinal schistosomiasis, *Schistosoma japonicum*, DNA-based molecular diagnostics, Real-time PCR, Neglected tropical diseases (NTDs)

## Abstract

**Background:**

Intestinal schistosomiasis, caused by infection with *Schistosoma japonicum*, remains a zoonotic neglected tropical disease (NTD) of significant public health importance in parts of China, the Philippines, and Indonesia. Ongoing schistosomiasis control programmes in these areas have achieved significant reductions in disease prevalence, transmission, and morbidity associated with infection; however, they have also presented new challenges, particularly in detecting and monitoring low-intensity and low-prevalence *S. japonicum* infections. Highly sensitive and highly specific diagnostic assays are therefore needed for effective disease diagnosis and transmission monitoring in low-endemicity areas, and the development of such assays has been recommended by the World Health Organisation.

**Methods:**

We first aimed to determine whether a *Schistosoma* spp. genus-specific real-time PCR assay routinely used to diagnose infections with African schistosome species (namely *Schistosoma mansoni* and *Schistosoma haematobium*), as well as two more recently developed *S. japonicum* species-specific real-time PCR assays, can accurately diagnose low-intensity *S. japonicum* infections. We also sought to optimise one of the *S. japonicum* species-specific real-time PCR assays by adding the same DNA extraction and PCR internal positive control target commonly used with the *Schistosoma* spp. genus-specific real-time PCR assay. This was done using in silico analyses and endpoint PCR with Sanger sequencing and real-time PCR using genomic DNA isolated from *Schistosoma* spp. adult worms, H_2_O spiked with individual *S. japonicum* ova, and naïve human faecal material also spiked with individual *S. japonicum* ova.

**Results:**

We demonstrate that the *Schistosoma* spp. genus-specific real-time PCR assay and one of the *S. japonicum* species-specific real-time PCR assays described here are likely incapable of reliably diagnosing low-intensity *S. japonicum* infections. We also demonstrate that the other *S. japonicum* species-specific real-time PCR assay can be performed using an additional DNA extraction and PCR internal positive control target and may reliably diagnose low-intensity *S. japonicum* infections.

**Conclusions:**

Whilst clinical assessment of the optimised *S. japonicum* species-specific real-time PCR assay described here is needed, it is our hope that this assay will become a standard and routinely used method to diagnose low-intensity *S. japonicum* infections and will be used support future *S. japonicum* transmission surveillance and elimination programmes.

**Graphical Abstract:**

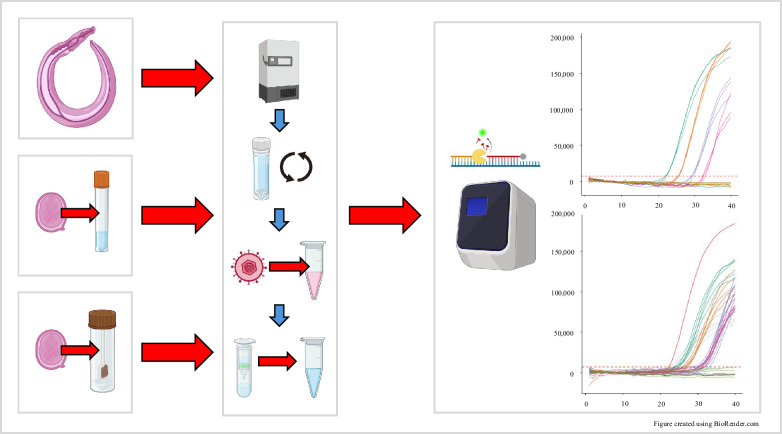

**Supplementary Information:**

The online version contains supplementary material available at 10.1186/s13071-026-07458-2.

## Background

Intestinal schistosomiasis caused by infection with the parasitic trematode *Schistosoma japonicum* remains a zoonotic neglected tropical disease (NTD) of significant public health importance in parts of China, the Philippines, and Indonesia [1, 2]. Ongoing schistosomiasis control programmes in these areas, first implemented in the mid-1980s and primarily involving the repeated mass distribution of the oral anthelmintic praziquantel, have achieved great reductions in disease prevalence, transmission and morbidity associated with infection [3–5]. However, despite their success, these ongoing control programmes have introduced new challenges, particularly in detecting and monitoring low-intensity and low-prevalence *S. japonicum* infections [6, 7].

Intestinal schistosomiasis is typically diagnosed using Kato-Katz faecal egg microscopy [8]. This method is relatively inexpensive and can be carried out at the point of care; however, it is considered low-throughput and lacks sensitivity, particularly when attempting to diagnose individuals harbouring low-intensity infections [9]. In addition, the morphology of *S. japonicum* ova is less distinctive than that of other parasitic helminths such as, for example, the primary *Schistosoma* species responsible for intestinal schistosomiasis in sub-Saharan Africa: *Schistosoma mansoni*. As a result, under microscopy, *S. japonicum* ova are commonly either missed or mistaken for other faecal pathogens and/or artefacts, and vice versa, leading to misdiagnosis [10, 11].

A variety of immunoassays have been developed to diagnose infection with intestinal *Schistosoma* species, the most widely used of which is the urine-based point-of-care circulating cathodic antigen (POC-CCA) assay [7, 12]. This assay, however, can also lack sensitivity and specificity, as it has frequently been reported that low-intensity *S. japonicum* infections are often missed by POC-CCA and that POC-CCA can provide false-positive results among individuals with urinary tract infections (UTIs), pregnant women, individuals demonstrating haematuria, and individuals infected with other trematode species endemic in parts of Asia, such as *Opisthorchis viverrini* [13, 14].

Whilst both Kato-Katz faecal egg microscopy and POC-CCA assays are valuable diagnostic tools in certain settings, highly sensitive DNA-based molecular assays, such as real-time PCR, are also needed for effective and impactful disease diagnosis and transmission monitoring in areas of low disease endemicity [15]. As such, their continued development and use have been recommended by the World Health Organisation (WHO) [16]. The most widely used and standardised real-time PCR assay to diagnose schistosomiasis targets a tandemly repeated genus-specific *Schistosoma* nuclear internal transcribed spacer 2 (ITS2) small subunit (SSU) ribosomal RNA (rRNA) region [17]. This assay has been used extensively to diagnose infection with *Schistosoma mansoni* in sub-Saharan Africa, as well as with *Schistosoma* species responsible for urogenital schistosomiasis across many parts of sub-Saharan Africa, including *Schistosoma haematobium* [7, 18]. It is also commonly performed as a duplex assay that incorporates an additional Phocid herpes virus-1 (PhHV-1) glycoprotein B DNA extraction and PCR internal positive control target locus [19–21]. This is primarily done to reduce false-negative diagnostic outcomes that may result from failed DNA isolation from clinical samples or failed PCR reactions rather than from an uninfected individual. However, whilst the *Schistosoma* ITS2 real-time PCR is highly sensitive for detecting *S. mansoni* and *S. haematobium* infections, it is not yet known whether this assay can accurately diagnose *S. japonicum* infections.

Additional real-time PCR assays targeting alternative DNA loci have been designed specifically to diagnose *S. japonicum* infection. These include assays that target a tandemly repeated species-specific satellite region [named the *Schistosoma japonicum* tandem repeat 1 (*Sj*TR1) region] [22] and a species-specific *S. japonicum* mitochondrial NADH dehydrogenase I (ND1) gene [25]. The former of these two assays has been used to diagnose infection with *S. japonicum* in humans and appears highly specific after having demonstrated no cross-reaction with other faecal parasites including *S. mansoni, Schistosoma mekongi*, nematode soil-transmitted helminths, cestodes, and various intestinal protozoan parasites [22]. However, to date, the *S. japonicum Sj*TR1 real-time PCR has been used only as a singleplex assay that does not incorporate a secondary DNA extraction and PCR internal positive control target. The latter of these two assays has also been used to diagnose infection with *S. japonicum* in humans [23–25], as well in cattle and water buffalo [23, 24], and also appears highly specific after having demonstrated no cross-reaction with other faecal helminths, including *S. mansoni, O. viverrini*, nematode soil-transmitted helminths, and pinworm [23–25]. In addition, the assay showed no cross reaction when challenged with gDNA isolated from adult-stage *S. haematobium* and *Schistosoma bovis* [25]. However, as with the *S. japonicum Sj*TR1 real-time PCR assay, the *S. japonicum* ND1 real-time PCR has been used only as a singleplex assay that does not incorporate a secondary DNA extraction and PCR internal positive control target. In addition, the sensitivity of this assay has not yet been assessed when clinical samples (i.e., faecal samples) have undergone homogenisation, which is now recommended when attempting to diagnose infections with faecal helminths using PCR, as homogenising faecal samples (typically using a ‘bead-beat’ approach) before DNA isolation can rupture helminth ova and liberate the internal genomic DNA (gDNA), increasing PCR sensitivity [26].

Here, we aimed to determine whether the genus-specific *Schistosoma* ITS2 real-time PCR assay (herein referred to as the ‘*Schistosoma* ITS2 real-time PCR assay’) can reliably diagnose low-intensity *S. japonicum* infections. In addition, we also aimed to assess the diagnostic performance of the *S. japonicum*-specific *Sj*TR1 real-time PCR assay (herein referred to as the ‘*S. japonicum Sj*TR1 real-time PCR assay’) and the *S. japonicum* ND1 real-time PCR assay (herein referred to as the ‘*S. japonicum* ND1 real-time PCR assay’). Following this, we sought to optimise the *S. japonicum* ND1 real-time PCR assay by adding the same PhHV-1 glycoprotein B DNA extraction and PCR internal positive control target commonly used with the *Schistosoma* ITS2 real-time PCR assay (when used to diagnose *S. mansoni* and *S. haematobium* infections) and assess its diagnostic performance. This was done in an attempt to develop a standardised methodology that can be used to reliably diagnose low-intensity *S. japonicum* infections using real-time PCR.

## Methods

### In silico specificity testing

The specificity of the *Schistosoma* ITS2, *S. japonicum Sj*TR1, and *S. japonicum* ND1 real-time PCR primers and probes was assessed in silico using *Schistosoma* sequence data downloaded from the National Center for Biotechnology Information [27] and WormBase ParaSite [28] online databases. When obtaining sequence data, care was taken to ensure that sequences were derived from a range of different strains within each *Schistosoma* species (see Additional file 1: Table S1).

#### *Schistosoma* ITS2 real-time PCR for *S. japonicum*

Three *S. japonicum* internal transcribed spacer 1 (ITS1)/5.8S/ITS2 sequences derived from China, two *S. japonicum* ITS2 sequences derived from the Philippines, and one *S. japonicum* 5.8S/ITS2/28S sequence derived from the Philippines were downloaded from the NCBI database. Three African *S. haematobium* ITS1/5.8S/ITS2 sequences and three African *S. mansoni* ITS1/5.8S/ITS2 sequences were also downloaded from the NCBI database (see Additional file 1: Table S1). These 12 sequences were aligned together with the *Schistosoma* ITS2 real-time PCR forward and reverse primers and probe sequences to assess any genetic variation at these regions that could have impacted PCR performance. This was done using a multiple sequence comparison by log-expectation (MUSCLE) alignment in Geneious Prime version 2026.0.2 (Dotmatics, LTD).

#### *Schistosoma japonicum Sj*TR1 real-time PCR

One *S. japonicum* *Sj*TR1 sequence derived from China was downloaded from the NCBI database [22]. As this was the only *Sj*TR1 sequence available, all *S. japonicum* whole-genome sequences were downloaded from the NCBI database and the WormBase ParaSite database (see Additional file 1: Table S1). All *Sj*TR1 target fragment sequences within each downloaded whole-genome sequence were identified and returned using *ecoPCR* (version 0.5.0; [29]). Forward and reverse primer sequences were allowed up to three base mismatches, and identified fragments were restricted to 60–100 bp in length. For all returned *Sj*TR1 fragments from each genome sequence, the fragment with the fewest primer base mismatches was selected and used for downstream multiple alignment. Returned fragments were aligned together with the downloaded *Sj*TR1 sequence as well as the *S. japonicum Sj*TR1 real-time PCR forward and reverse primer and probe sequences to again assess any genetic variation at these regions that could have impacted PCR performance. This was done as described above.

#### *Schistosoma japonicum* ND1 real-time PCR

Three *S. japonicum* whole mitochondrial genome sequences derived from China and three *S. japonicum* whole mitochondrial genome sequences derived from the Philippines were downloaded from the NCBI database. Three African *S. haematobium* whole mitochondrial genome sequences, one African *S. mansoni* whole mitochondrial genome sequence, and two African *S. mansoni* mitochondrial ND1 sequences were also downloaded from the NCBI database (see Additional file 1: Table S1). These 12 sequences were aligned together with the *S. japonicum* ND1 real-time PCR forward and reverse primers and probe sequences to again assess any genetic variation at these regions that could have impacted PCR performance. This was again done as described above.

### *Schistosoma* material used during real-time PCR assay optimisation and analytical assessment

#### *Schistosoma* adult worms

Genomic DNA (gDNA) was isolated from whole *S. japonicum*, *S. mansoni*, and *S. haematobium* adult worms using a standard protocol (see Additional file 2: Text S1 [30]). The DNA concentration of each isolate was then measured using a Nanodrop spectrophotometer (Thermo Fisher Scientific, Waltham, MA, USA) and normalised to a concentration of 1 ng/µl using nuclease-free H_2_O. The species of each isolate was confirmed using the protocol outlined in Additional file 3: Text S1). Details of *Schistosoma* adult gDNA material, including sample IDs, suppliers, laboratory strain, and DNA extraction method, are outlined in Table 1.

**Table 1:** *Schistosoma* spp. material used during *Schistosoma japonicum* ND1 real-time PCR optimisation and analytical assessment

Sample ID	Specimen	Collection location or laboratory strain	DNA extraction method	Provided by
*Sj*	*S. japonicum* adult worms (*n* = 3, collectively extracted; 1 ng/µl)	Laboratory passaged: Chinese strain	DNeasy blood and tissue kit (QIAGEN, UK) following a standard protocol (see Additional file 2: Text S1*,* [32])	SCAN*
*Sm*	*S. mansoni* adult worms (*n* = 3, collectively extracted; 1 ng/µl)	Laboratory passaged: Senegal strain		BRI*
*Sh*	*S. haematobium* adult worms (*n* = 3, collectively extracted; 1 ng/µl)	Laboratory passaged, Egyptian strain		SSR*
*Sj*-10egg (*n* = 5)	*S. japonicum* ova (*n* = 10 ova, collectively extracted; *n* = 5 replicates)	Laboratory passaged: Chinese strain	DNeasy blood and tissue kit (QIAGEN, UK) following a standard protocol with minor revisions as described above (see Additional file 4: Text S1 [32])	BRI*
*Sj*-5egg (*n* = 5)	*S. japonicum* ova (*n* = 5 ova, collectively extracted; *n* = 5 replicates)			
*Sj-*1egg (*n* = 10)	*S. japonicum* ova (*n* = 1 ova, individually extracted; *n* = 10 replicates)			
*Sj-*1egg-stool (*n* = 5)	*S. japonicum* ova (*n* = 1 ova, individually extracted; *n* = 5 replicates)		DNeasy blood and tissue kit (QIAGEN, UK) following a standard protocol with minor revisions as described above (see Additional file 5: Text S1 [19])	Ova provided by BRI*
Naïve_stool (*n* = 5)	Not applicable	Not applicable		Not applicable

*SCAN: Schistosome Collections at the Natural History Museum, London, UK [33], BRI: Biomedical Research Institute (BRI), Rockville, MD, USA [31], SSR: Schistosome and Snail Resource, London, UK [34]

#### H_2_O spiked with ***S. japonicum*** ova

Viable *S. japonicum* ova were sourced from the Biomedical Research Institute (BRI), Rockville, MD, USA [31]. All ova were delivered to the Natural History Museum, London, UK, chilled, and immediately quantified upon arrival using light microscopy. A total of 20 µl nuclease-free H_2_O was spiked with either 10, 5, or 1 single *S. japonicum* ova under a dissecting microscope using a Gilson pipette (Gilson, UK). Genomic DNA was then isolated from all H_2_O ova-spiked samples, as well as two DNA extraction-negative control samples that were subjected to the same DNA extraction process but were not spiked with *S. japonicum* ova following a standard protocol with minor revisions (see Additional file 4: Text S1 [32]). These included: (i) adding a bead-beating step using an MPBio FastPrep-24 5G cell disrupter and 0.45 g of 1.4-mm ceramic beads (MPBiomedicals, USA; 30 s; 20 Hertz p/second agitation frequency) and (ii) adding 2 µl of a Phocid Herpes Virus-1 (PhHV-1) working solution [a stock PhHV-1 culture supernatant solution (European Virus Archive global (EVAg), France) was diluted 1:100 using nuclease-free H_2_O to make up a PhHV-1 working solution] to the tissue lysis buffer/Proteinase K mix prior to tissue lysis to act as a DNA extraction and PCR internal positive control [19]. Details of all H_2_O ova-spiked samples, including number of replicates, sample IDs, suppliers, *Schistosoma* ova laboratory strains, and DNA extraction method, are outlined in Table 1.

#### Naïve faecal material spiked with *S. japonicum* ova

Human faecal material provided by a donor from an area non-endemic for any form of schistosomiasis was filtered using a standard 212-µM gauge filter. A total of 0.2 g filtered faecal material was then transferred to a labelled 2-ml screwcap tube. This was repeated nine times to make up ten 0.2-g faecal sample replicates. Five of these samples were then spiked with one single *S. japonicum* ova using a Gilson pipette (Gilson, UK). DNA was isolated from all faecal samples (ova-spiked and non-spiked) as well as two DNA extraction-negative control samples that were subjected to the same DNA extraction process but did not contain any faecal material or *Schistosoma* ova following a standard protocol with minor revisions (see Additional file 5: Text S1, [19]). These included: (i) subjecting all faecal samples to a snap-freeze step, whereby each sample was homogenised in 250 µl of a 2% polyvinylpolypyrrolidone (PVPP)/phosphate buffered saline (PBS) suspension, stored at − 80 °C for 45 min, and then thawed at ambient temperature for 45 min to weaken the outer layer of helminth ova prior to the subsequent bead-beating step; (ii) adding a bead-beating step using an MPBio FastPrep-24 5G cell disrupter and 0.9 g of 1.4-mm ceramic beads (MPBiomedicals, USA; 30 s; 20 Hertz p/second agitation frequency); (iii) adding 2 µl of a Phocid Herpes Virus-1 (PhHV-1) working solution as above to act as a DNA extraction and PCR internal positive control [19]. Details of all faecal samples (ova-spiked and non-spiked), including sample IDs, *S. japonicum* ova supplier, *S. japonicum* ova laboratory strain, and DNA extraction method, are outlined in Table 1.

### Temperature gradient endpoint PCR and Sanger sequencing to confirm real-time PCR target DNA loci and determine optimal real-time PCR annealing temperatures

To confirm amplification of target DNA loci for the *Schistosoma* ITS2 real-time PCR assay, the *S. japonicum Sj*TR1 real-time PCR assay, and the *S. japonicum* ND1 real-time PCR assay, three temperature gradient endpoint PCRs were performed followed by Sanger sequencing, nucleotide Basic Local Alignment Search Tool (BLASTn) analysis within the National Centre for Biotechnology Information (NCBI) database [27], and pairwise alignment of sequence data to reference sequence data. Details of all endpoint PCR reaction mixes, temperature gradient PCR conditions, amplicon purification, Sanger sequencing, sequence data analysis/editing, and BLASTn analysis/sequence alignment are described in Additional file 3*:* Text S2.

### Optimisation and analytical assessment of a TaqMan™ probe-based diagnostic real-time PCR assay that incorporates an additional DNA extraction and PCR internal positive control

#### Assessment of real-time PCR primer/probe concentrations and annealing temperatures

*Schistosoma* ITS2 real-time PCR for *S. japonicum*

To assess the optimal real-time PCR primer/probe concentrations and annealing temperature of the *Schistosoma* ITS2 real-time PCR (for *S. japonicum,* specifically), multiple real-time PCR assays were performed using previously designed forward (*Ssp_ITS_48_FW*) and reverse (*Ssp_ITS_124_RV)* primers and a previously designed probe *(Ssp_ITS_78T_Pro)* [17] (Table 2).

**Table 2:** Primer and modified probe oligonucleotide sequences used to amplify a 77-bp fragment of the genus-specific *Schistosoma* spp. internal transcribed spacer 2 (ITS2) ribosomal DNA (rDNA) region

Name	Target	Oligonucleotide sequence (5′–3′)	Reference
Ssp_ITS_48_FW*	*Schistosoma* spp. ITS2	GGTCTAGATGACTTGATYGAGATGCT	[17]
Ssp_ITS_124_RV^†^		TCCCGAGCGYGTATAATGTCATTA	
Ssp_ITS_78T_Pro^‡^		(FAM^¥^) TGGGTTGTGCTCGAGTCGTGGC/3IABkFQ^¶^/	

*Forward primer

^†^Reverse primer

^‡^Probe

^¥^Fluorophore

^¶^Iowa Black Dark FQ quencher

All *Schistosoma* ITS2 qPCRs were carried out using 25-µl qPCR reactions made up of 4 µl *Sj* template DNA that had been normalised to a concentration of 0.25 ng/µl using nuclease-free H_2_O, various primer/probe concentration combinations performed in triplicate (see Additional file 3: Text S3 and Table S5), varying volumes of nuclease-free H_2_O (depending on the volume of primers and probe added), and 12.5 µl Luna Universal Probe qPCR Master mix (NEB, USA). Initially, the following cycling conditions were used: 10 min at 95 °C; 40 cycles of 15 s at 95 °C and 60 s at 60 °C. This was then repeated using annealing temperatures of 58 and 56 °C. All real-time PCR reactions were performed using a StepOne qPCR system thermocycler (Thermo Fisher Scientific, USA). Real-time PCR reactions were considered positive for the presence of *S. japonicum* DNA if amplificated curves [normalised using a ROX reference dye incorporated into the Luna Universal Probe qPCR Master mix (NEB, USA)] crossed a fluorescence threshold determined by the StepOnePlus software (default settings; software version 2.3; Thermo Fisher Scientific, USA) and followed a smooth, sigmoid amplification profile typical of real-time PCR amplification curves. No further testing took place using the *Schistosoma* ITS2 real-time PCR assay.

*Schistosoma japonicum* SjTR1 real-time PCR

To assess the optimal real-time PCR primer/probe concentrations and annealing temperature of the *S. japonicum Sj*TR1 real-time PCR, multiple real-time PCR assays were performed using previously designed forward (Sj*TR1_FW*) and reverse (Sj*TR1_RV*) primers and a previously designed probe (Sj*TR1_Pro*) [22] (Table 3).

**Table 3:** Primer and probe oligonucleotide sequences used to amplify a *Schistosoma japonicum* 80-bp tandemly repeated region [22]

Name	Target	Oligonucleotide sequence (5′–3′)	Reference
*Sj*TR1_FW*	*S. japonicum Sj*TR1 locus	TGTCGTGCACAACCTTCTTC	[22]
*Sj*TR1_RV^†^		ACAACTCATCACCGCCAATC	
*Sj*TR1_Pro^‡^		(FAM^¥^) TGGCGAGAT/ZEN^⁒^/ GTTGTGGGTGTAAGT/3IABkFQ^¶^/	

*Forward primer

^†^Reverse primer

^‡^Probe

^¥^Fluorophore

^⁒^Additional ZEN quencher [22]

^¶^Iowa Black Dark FQ quencher

All *S. japonicum Sj*TR1 real-time PCRs were carried out using 25-µl qPCR reactions made up of 4 µl *Sj* template DNA that had been normalised to a concentration of 0.25 ng/µl using nuclease-free H_2_O, various primer/probe concentration combinations performed in triplicate (see Additional file 3: Text S3 and Table S6), varying volumes of nuclease-free H_2_O (depending on the volume of primers and probe added), and 12.5 µl Luna Universal Probe qPCR Master mix (NEB, USA). Initially, the following cycling conditions were used: 10 min at 95 °C; 40 cycles of 15 s at 95 °C and 60 s at 59 °C. This was then repeated using annealing temperatures of 54, 55, 56, 57, 58, 60, and 61 °C. All real-time PCR reactions were performed using a StepOne qPCR system thermocycler (Thermo Fisher Scientific, USA). Real-time PCR reactions were considered positive for the presence of *S. japonicum* DNA as described above. No further testing took place using the *S. japonicum* SjTR1 real-time PCR assay.

*Schistosoma japonicum* ND1 real-time PCR

To assess the optimal real-time PCR primer/probe concentrations and annealing temperature of the *S. japonicum* ND1 real-time PCR, multiple real-time PCR assays were performed using previously designed forward (*Sj_ND1_FW*) and reverse (*Sj_ND1_RV*) primers and a previously designed probe (*Sj_ND1_Pro*) [25] (Table 4).

**Table 4:** Primer and probe oligonucleotide sequences used to amplify a 75-bp fragment of the *Schistosoma japonicum* mitochondrial ND1 gene and an 89-bp fragment of the Phocid herpes virus-1 (PhHV-1) glycoprotein B gene using PCR and real-time PCR

Name	Target	Oligonucleotide sequence (5′–3′)	References
Sj_ND1_FW*	*S. japonicum* mitochondrial ND1 locus (diagnostic target)	ACTGGTTATGGTTTGTTGATGTTAGGT	[25]
Sj_ND1_RV^†^		AGCCACACGAACAGCACTAATC	
Sj_ND1_Pro^‡^		(FAM^¥^) AGGTTCTTGGAAAAAGTAT/3IABkFQ^⁋^/	
PhHV-1_267S_FW*	PhHV-1 glycoprotein B (internal control target)	GGGCGAATCACAGATTGAATC	[19, 35, 36]
PhHV-1_337AS_RV^†^		GCGGTTCCAAACGTACCAA	
PhHV-1_305TQ_Pro^‡^		(SUN^¥^) TTTTTATGTGTCCGCCACCATCTGGATC/3IABkFQ^¶^/	

*Forward primer

^†^Reverse primer

^‡^Probe

^¥^Fluorophore

^¶^Iowa Black Dark FQ quencher

All *S. japonicum* ND1 real-time PCRs were carried out using 25-µl qPCR reactions made up of 4 µl *Sj* template DNA that had been normalised to a concentration of 0.25 ng/µl using nuclease-free H_2_O, various primer/probe concentration combinations performed in triplicate (see Additional file 3: Text S3 and Table S7), varying volumes of nuclease-free H_2_O (depending on the volume of primers and probe added), and 12.5 µl Luna Universal Probe qPCR Master mix (NEB, USA). Initially, the following cycling conditions were used: 10 min at 95 °C; 40 cycles of 15 s at 95 °C and 60 s at 60 °C. This was then repeated using annealing temperatures of 59, 58, 57, and 56 °C. All real-time PCR reactions were performed using a StepOne qPCR system thermocycler (Thermo Fisher Scientific, USA). Real-time PCR reactions were considered positive for *S. japonicum* DNA as described above. *Schistosoma japonicum* ND1 real-time PCR primer and probe concentrations of 600 nM and 500 nM, respectively, and an annealing temperature of 56 °C were used for all further testing.

#### Analytical sensitivity testing: serially diluted *S. japonicum* adult worm gDNA

The analytical sensitivity of the *S. japonicum* ND1 real-time PCR was tested using 1 ng, 0.1 ng, 0.01 ng, 1 pg, 0.1 pg, 0.01 pg, and 1 fg of *S. japonicum* gDNA. This was done by normalising *Sj* template DNA (1 ng/µl) to a concentration of 0.25 ng/µl using nuclease-free H_2_O. This 0.25 ng/µl sample was then serially diluted 1:10 through six steps using nuclease-free H_2_O. The *S. japonicum* ND1 real-time PCR assay was then performed as described above using each *S. japonicum* gDNA concentration in triplicate and one no-template negative control using nuclease-free H_2_O in place of template DNA.

#### In vitro specificity testing

To further test for *S. japonicum* ND1 target loci cross-reactivity with closely related *Schistosoma* species *S. mansoni* and *S. haematobium* [37], the *S. japonicum* ND1 real-time PCR assay was performed as described above using *Sj, Sm*, and *Sh* gDNA that had been normalised to a concentration of 0.25 ng/µl using nuclease-free H_2_O in triplicate and one no-template negative control using nuclease-free H_2_O in place of template DNA.

#### Addition of a phocid herpes virus-1 (PhHV-1) glycoprotein B target to the *S. japonicum* ND1 real-time PCR to act as a DNA extraction and PCR internal positive control

The *S. japonicum* ND1 real-time PCR assay was performed as described above with additional previously designed forward (*PhHV-1_267S_FW*) and reverse (*PhHV-1_337AS_RV*) primers and a previously designed probe (*PhHV-1_305TQ_Pro*) [19, 35, 36] (Table 4). The *S. japonicum* ND1 real-time PCR was therefore carried out using 25-µl qPCR reactions made up of 4 µl template DNA, 3.75 nuclease-free H_2_O, 600 nM (1.5 µl of a 10 µmol working solution) *S. japonicum* ND1 forward (*Sj_ND1_FW*) and reverse (*Sj_ND1_*RV) primers and 500 nM (1.25 µl of a 10 µmol working solution) *S. japonicum* ND1 probe (*Sj_ND1_Pro*), 60 nM (0.15 µl of a 10 µmol working solution) PhHV-1 glycoprotein B forward (*PhHV-1_267S_FW*) and reverse (*PhHV-1_337AS_RV*) primers and 100 nM (0.25 µl of a 10 µmol working solution) PhHV-1 glycoprotein B probe, and 12.5 µl Luna Universal Probe qPCR Master mix (NEB, USA). It was performed using all five *Sj-*10egg samples (Table 1) in triplicate, one no-template negative control with nuclease-free H_2_O in place of template DNA, both DNA-extraction-negative control samples, and one positive control with *Sj* gDNA. Initially, the following cycling conditions were used: 10 min at 95 °C; 40 cycles of 15 s at 95 °C and 60 s at 56 °C. This was then repeated using an annealing temperature of 60 °C so that comparisons could be made between PhHV-1 glycoprotein B target amplification curves when using annealing temperatures of 56 °C and 60 °C (the annealing temperature used when performing the *Schistosoma* ITS2 real-time PCR with the additional PhHV-1 glycoprotein B target and using the Luna Universal Probe qPCR Master mix (NEB, USA) [19]). Both *S. japonicum* ND1 and PhHV-1 glycoprotein B targets were considered positive, as described above. The additional PhHV-1 glycoprotein B target was incorporated into all further *S. japonicum* ND1 real-time PCR testing.

#### Analytical sensitivity testing: H_2_O spiked with ***S. japonicum*** ova and PhHV-1

The ability of the *S. japonicum* ND1 real-time PCR to detect *S. japonicum* gDNA isolated from 10, 5, and 1 single *S. japonicum* ova, as well as the PhHV-1 DNA extraction and PCR internal positive control target, was tested using all *Sj*-10egg, *Sj*-5egg, and *Sj*-1egg gDNA samples, one no-template negative control using nuclease-free H_2_O in place of template DNA, both DNA extraction-negative control samples, and one positive control using *Sj* gDNA. The *S. japonicum* ND1 real-time PCR was performed as described above. PhHV-1 glycoprotein B amplification curves were scrutinised across all reactions to assess whether the concentration of *S. japonicum* gDNA, which differed across *Sj*-10egg, *Sj*-5egg, and *Sj*-1egg gDNA samples, affected amplification curve profiles.

#### Analytical sensitivity testing: Naïve faecal material spiked with *S. japonicum* ova and PhHV-1

The ability of the *S. japonicum* ND1 real-time PCR to detect *S. japonicum* gDNA isolated from 0.2 g faecal material spiked with one single *S. japonicum* ova, as well as the PhHV-1 DNA extraction and PCR internal positive control target, was tested using all faecal DNA samples (ova-spiked and non-spiked), one no-template negative control using nuclease-free H_2_O in place of template DNA, both DNA extraction-negative control samples, and one positive control using *Sj* gDNA. PhHV-1 glycoprotein B amplification curves were scrutinised across all reactions and compared with those produced during analytical sensitivity testing using H_2_O spiked with *S. japonicum* ova to assess whether the presence of faecal material affected amplification curve profiles.

#### Confirming the presence of *S. japonicum* DNA in faecal DNA samples

To confirm the presence of *S. japonicum* DNA in faecal DNA samples, *S. japonicum* ND1 endpoint PCR followed by Sanger sequencing and BLASTn analysis/alignment to a *S. japonicum* whole mitochondrial genome sequence was carried out using all faecal DNA samples (ova-spiked and non-spiked), one no-template negative control using nuclease-free H_2_O in place of template DNA, and one positive control using *Sj* gDNA. This was done as with the temperature gradient endpoint PCR outlined above; however, only one annealing temperature (56 °C) was used.

## Results

### In silico specificity testing

#### Schistosoma ITS2 real-time PCR for *S. japonicum*

MUSCLE alignment revealed five single-nucleotide polymorphisms (SNPs) in the *Schistosoma* ITS2 real-time PCR forward primer region between African and Asian schistosome species and one SNP in the probe region between these species. In addition, four SNPs, as well as a 7-bp indel, were identified in the reverse primer region between these species (see Additional File 1*:* Fig. S1).

#### *Schistosoma japonicum* SjTR1 real-time PCR

Four *S. japonicum* whole-genome sequences derived from China were downloaded from the NCBI database and three *S. japonicum* whole-genome sequences derived from China were downloaded from the WormBase ParaSite database (see Additional File 1*:* Table S1). Notably, no Philippines-derived *S. japonicum* whole-genome sequence data was available. The *Sj*TR1 target fragment was successfully identified and returned from four of these seven whole-genome sequences. MUSCLE alignment revealed two SNPs in the *S. japonicum Sj*TR1 real-time PCR forward primer region in one of these four returned fragments and no SNPs in the reverse primer region across any of the returned fragments. In addition, one SNP was identified in the probe region in three of these four returned fragments, a single but different SNP was identified in the probe region in the other returned fragment, and multiple SNPs, as well as an indel, were identified in one of the returned fragments (see Additional File 1*:* Fig. S2).

#### *Schistosoma japonicum* ND1 real-time PCR

MUSCLE alignment revealed eight SNPs in the *S. japonicum* ND1 real-time PCR forward primer region between African and Asian schistosome species, with additional SNPs in the forward primer region between *S. haematobium* and Asian schistosome species and *S. mansoni* and Asian schistosome species. A further five SNPs were identified in the probe region between African and Asian schistosome species, with additional SNPs in the probe region between *S. haematobium* and Asian schistosome species and *S. mansoni* and Asian schistosome species. In addition, five SNPs were identified in the reverse primer region between African and Asian schistosome species, with additional SNPs in the reverse primer region between *S. mansoni* and Asian schistosome species. Notably, no SNPs were identified between any Asian *S. japonicum* species (inclusive of three Chinese and three Philippine sequences) across the entire 75-bp target locus, including within the forward and reverse primer and probe regions (see Additional File 1*:* Fig. S3).

### Temperature gradient endpoint PCR and Sanger sequencing to confirm real-time PCR target DNA loci and determine optimal real-time PCR annealing temperatures

#### Schistosoma ITS2 endpoint PCR for *S. japonicum*

When using the Luna Universal Probe qPCR Master mix [New England Biolabs (NEB), USA], a ~ 77-bp amplicon was produced in all three replicates when using annealing temperatures of 60 °C, 59 °C, and 58 °C. All nine of these amplicons were visible but of poor quality when examined using gel electrophoresis, suggesting sub-optimal amplification. No amplicons were produced at annealing temperatures of 56 °C or 57 °C. This was also the case when using Illustra PuReTaq ready-to-go PCR beads (Sigma-Aldrich, USA) instead of the Luna Universal Probe qPCR Master mix (NEB, USA). Sanger sequence data were generated from all 18 amplicons (nine from each PCR method); however, all sequence chromatograms were of poor quality and so could not be reliably confirmed as the *S. japonicum* ITS2 target sequence using BLASTn analysis, nor through alignment to the reference *S. japonicum* 18S/ITS1/5.8S/ITS2/28S rDNA reference sequence.

#### *Schistosoma japonicum* SjTR1 endpoint PCR

No amplicons were produced across all annealing temperatures when using both the Luna Universal Probe qPCR Master mix (New England Biolabs (NEB), USA) and Illustra PuReTaq ready-to-go PCR beads (Sigma-Aldrich, USA).

#### Schistosoma japonicum ND1 endpoint PCR

All 15 endpoint PCR reactions (all three reactions tested at all five annealing temperatures) produced a ~ 75-bp amplicon. Sanger sequence data were successfully obtained from all 15 amplicons, all of which were identical and confirmed as the *S. japonicum* ND1 target sequence using BLASTn analysis and through alignment to the reference *S. japonicum* mitochondrial genome. No amplification was observed in the H_2_O negative control. The generated 75-bp *S. japonicum* ND1 target locus sequence (5′–3′ orientation) is shown in Additional file 1: Fig. S3 and found in FASTA format in Additional file 6: Dataset S1*.*

### Optimisation and analytical assessment of a TaqMan™ probe-based diagnostic real-time PCR assay that incorporates an additional DNA extraction and PCR internal positive control target locus

#### Assessment of various real-time PCR primer/probe concentrations and annealing temperatures

##### *Schistosoma* ITS2 real-time PCR for *S. japonicum*

Performing the *Schistosoma* ITS2 real-time PCR using 100-nM forward and reverse primers and probe, with an annealing temperature of 60 °C, was deemed optimal compared with all other real-time PCR conditions. When doing so, real-time PCR amplification curves showed the greatest fluorescence value and lowest quantification cycle (*C*_*q*_) value (*C*_*q*_ ~ 37), whilst following a smooth, sigmoidal amplification profile that was consistent across all three replicates. In addition, under these conditions, no amplification was observed in the H_2_O negative control.

##### *Schistosoma japonicum* SjTR1 endpoint PCR

No amplification curves were produced across all primer/probe concentrations and annealing temperatures tested.

##### *Schistosoma japonicum* ND1 real-time PCR

Performing the *S. japonicum* ND1 real-time PCR using 600 nM forward and reverse primers and 500 nM probe, with an annealing temperature of 56 °C, was deemed optimal. When doing so, real-time PCR amplification curves showed the greatest fluorescence value and lowest *C*_*q*_ value (*C*_*q*_ ~ 22), whilst following a smooth, sigmoidal amplification profile that was consistent across all three replicates. In addition, under these conditions, no amplification was observed in the H_2_O negative control.

#### Analytical sensitivity testing: serially diluted *S. japonicum* adult worm gDNA

The *S. japonicum* ND1 real-time PCR produced an amplification curve in all 1 ng, 0.1 ng, 0.01 ng, and 1 pg replicates. As *S. japonicum* gDNA concentrations decreased, *S. japonicum* ND1 real-time PCR *C*_*q*_ values increased, as anticipated. No amplification curve was produced in any 0.01 pg, 0.01 pg, or 1 fg replicates. The real-time PCR standard curve yielded a slope of − 3.3493, a *y*-intercept of 42.73, an R^2^ value of 0.997, and a calculated amplification efficiency of 93.3%. All *S. japonicum* ND1 real-time PCR analytical sensitivity data are described in Table 5. All *S. japonicum* ND1 real-time PCR data concerning serially diluted *S. japonicum* adult worm gDNA are shown in Additional file 7*:* Fig. S1. No amplification was observed in the H_2_O negative control.

**Table 5:** *Schistosoma* ITS2 real-time PCR and *Schistosoma japonicum* ND1 real-time PCR sensitivity data

Sample ID	gDNA concentration	*n* replicates	% Positive by *S. japonicum* ND1 real-time PCR	Mean *S. japonicum* ND1 real-time PCR *C*_*q*_ value ± SD^‡^
*Sj*	1 ng	3*	100	21.99 ± 0.16
	0.1 ng	3*	100	24.91 ± 0.06
	0.01 ng	3*	100	28.82 ± 0.57
	1 pg	3*	100	32.33 ± 0.65
	0.1 pg	3*	0	Not applicable
	0.01 pg	3*	0	Not applicable
	1 fg	3*	0	Not applicable
*Sj-*10egg	Not applicable	5^†^	100	24.73 ± 0.26
*Sj-*5egg	Not applicable	5^†^	100	26.62 ± 0.55
*Sj-*1egg	Not applicable	10^†^	100	31.23 ± 1.28
*Sj-*1egg-stool	Not applicable	5^†^	80	31.67 ± 0.97
naïve_stool	Not applicable	5^†^	0	Not applicable

^*^Replicate samples from the same gDNA isolates

^†^Replicates using different DNA isolates

^‡^Standard deviation

#### In vitro specificity testing

The *S. japonicum* ND1 real-time PCR produced an amplification curve in all *Sj* gDNA replicates. No non-target amplification was observed in any *Sm* or *Sh* gDNA replicates. No amplification was observed in the H_2_O negative control.

#### Addition of a phocid herpes virus-1 (PhHV-1) glycoprotein B target to the *S. japonicum* ND1 real-time PCR to act as a DNA extraction and PCR internal positive control

A PhHV-1 glycoprotein B real-time PCR amplification curve was produced when using all *Sj*-10egg samples, in all replicates, when using annealing temperatures of 56 °C and 60 °C. PhHV-1 glycoprotein B amplification curve *C*_*q*_ values and profiles were similar across all samples and so did not appear to be affected by reducing the annealing temperature from 60 to 56 °C. All real-time PCR controls performed as anticipated.

#### Analytical sensitivity testing: H_2_O spiked with ***S. japonicum*** ova and PhHV-1

The *S. japonicum* ND1 real-time PCR produced an amplification curve in all *Sj-*10egg, *Sj*-5egg, and *Sj-*1egg replicates. As the number of ova decreased, *S. japonicum* ND1 real-time PCR *C*_*q*_ values increased, as anticipated. An ND1 amplification curve was not produced in either of the DNA extraction-negative control samples. A PhHV-1 glycoprotein B amplification curve was produced in all *Sj-*10egg, *Sj*-5egg, and *Sj-*1egg replicates and in both DNA extraction-negative control samples. PhHV-1 glycoprotein B amplification curve *C*_*q*_ values and profiles were similar across all samples and so did not seem to be affected by the different concentrations of *S. japonicum* gDNA. ND1 real-time PCR data for H_2_O spiked with *S. japonicum* ova and PhHV-1 are shown in Additional file 7: Fig. S2. All real-time PCR controls performed as anticipated.

#### Analytical sensitivity testing: Naïve faecal material spiked with *S. japonicum* ova and PhHV-1

The *S. japonicum* ND1 real-time PCR produced an amplification curve in four of the five (80%) *Sj*-1egg-stool DNA samples. No amplification was observed in one of the five (20%) *Sj*-1egg-stool DNA samples or in any of the ‘naïve_stool’ samples. In addition, a PhHV-1 glycoprotein B amplification curve was produced in all *Sj*-1egg-stool samples, in all ‘naïve_stool’ samples, and in both extraction negative control samples. PhHV-1 glycoprotein B amplification curve *C*_*q*_ values and profiles were similar across all samples. ND1 real-time PCR data concerning naïve faecal material spiked with *S. japonicum* ova and PhHV-1 are shown in Additional file 7*:* Fig. S2. All real-time PCR controls performed as anticipated.

#### Confirming the presence of *S. japonicum* DNA in faecal DNA samples

The four *Sj*-1egg-stool DNA samples that were positive using the *S. japonicum* ND1 real-time PCR produced a ~ 75 bp amplicon during *S. japonicum* ND1 endpoint PCR testing. No amplification was observed in the one *Sj*-1egg-stool DNA sample that was negative using *S. japonicum* ND1 real-time PCR, or in any of the ‘naïve_stool’ samples, during *S. japonicum* ND1 endpoint PCR testing. All real-time PCR controls performed as anticipated. Forward and reverse Sanger sequence data were successfully obtained from all four *Sj*-1egg-stool DNA samples, all of which were identical and confirmed as the *S. japonicum* ND1 target sequence using BLASTn analysis and through alignment to the reference *S. japonicum* mitochondrial genome.

## Discussion

Given the many successes of schistosomiasis control programmes implemented throughout Asia since the 1980s, highly sensitive methods of detecting any remaining and low-intensity *S. japonicum* infections are now needed to eliminate intestinal schistosomiasis caused by *S. japonicum* in this area [6]. Here, we aimed to determine whether the *Schistosoma* ITS2 real-time PCR assay [17], often used to diagnose *S. mansoni* and *S. haematobium* infections in sub-Saharan Africa, can reliably diagnose low-intensity *S. japonicum* infections. In addition, we aimed to assess the diagnostic performance of the previously developed *S. japonicum*-specific *Sj*TR1 real-time PCR assay [22] and the previously developed *S. japonicum* ND1 real-time PCR assay [25]. Following this, we sought to optimise the *S. japonicum* ND1 real-time PCR assay by adding the same PhHV-1 glycoprotein B DNA extraction and PCR internal positive control target commonly used with the *Schistosoma* ITS2 real-time PCR assay (when used to diagnose *S. haematobium* and *S. mansoni* infections) and assess its diagnostic performance. This was done to develop a standardised methodology that can be used to reliably diagnose low-intensity *S. japonicum* infections using real-time PCR.

### Diagnostic performance of the *Schistosoma* ITS2 real-time PCR for *S. japonicum*

In silico analysis suggested that the *Schistosoma* ITS2 real-time PCR forward and reverse primers are unlikely to anneal efficiently, if at all, to *S. japonicum* template DNA. Moreover, during in vivo analysis, whilst visible, the amplicons of the correct length produced during *Schistosoma* ITS2 endpoint PCR testing were of poor quality and could not be confirmed as the assay’s target locus as all generated sequence data wwew also of poor quality. This was the case when using the Luna Universal Probe qPCR Master Mix [New England Biolabs (NEB), USA; commonly used when performing *Schistosoma* ITS2 real-time PCR testing for African schistosome species] and when using one Illustra PuReTaq ready-to-go PCR bead (Sigma-Aldrich, USA); these have previously been successfully used to amplify and Sanger sequence the same ITS2 locus in both *S. mansoni* and *S. haematobium* using the same approach as part of a different study.

Performing the *Schistosoma* ITS2 real-time PCR using 100 nM forward and reverse primers and probe, with an annealing temperature of 60 °C, was deemed optimal compared with all other real-time PCR conditions. However, even under these optimal conditions and when using 1 ng *S. japonicum* gDNA, the *Schistosoma* ITS2 real-time PCR produced amplification curves with a quantification cycle (*C*_*q*_) value of *C*_*q*_ ~ 37. By comparison, when used with 1 ng *S. mansoni* gDNA and 1 ng *S. haematobium* gDNA, the *Schistosoma* ITS2 real-time PCR assay consistently produces amplification curves with *C*_*q*_ values of ~ 29, which are comparatively much lower than those produced when using 1 ng *S. japonicum* gDNA. These findings suggest that the *Schistosoma* ITS2 real-time PCR assay may fail to reliably detect low-intensity *S. japonicum* infections, likely because of ITS2 sequence variation between Asian and African species.

### Diagnostic performance of the *S. japonicum* SjTR1 real-time PCR

In silico analysis suggested that the *S. japonicum Sj*TR1 forward and reverse primers may also be unable to anneal efficiently, if at all, to *S. japonicum* template DNA, given that the *Sj*TR1 target locus was detected in only four of the seven whole-genome sequences. Additionally, multiple SNPs and a single-base indel were observed in the probe region of the returned target fragments, indicating poor probe specificity across isolates. Notably, whilst all of our reference whole-genome sequences were derived from China (no whole-genome data from outside of China, e.g., the Philippines, were available), the initial design of this assay was based on *S. japonicum* whole-genome data also derived from China [22]. Furthermore, during in vitro analysis, we were unable to successfully amplify the *Sj*TR1 locus using both endpoint and real-time PCR despite using a wide range of primer/probe concentrations and annealing temperatures, and despite our *S. japonicum* material (adult worms and ova) being derived from China. Consequently, no further evaluation or optimisation of this assay was possible.

### Diagnostic performance of the *S. japonicum* ND1 real-time PCR

No SNPs were present in Chinese and Philippine *S. japonicum* sequences across the entire 75-bp *S. japonicum* ND1 target locus during in silico analysis, including at the forward, reverse, and probe binding sites, despite known mitochondrial genetic diversity between these two groups [38]. This was also the case for the *S. japonicum* ND1 target fragment sequence generated here using *S. japonicum* material derived from China. It was found that the optimised *S. japonicum* ND1 real-time PCR assay consistently produced an amplification curve when used with as little as 1 pg of *S. japonicum* gDNA (derived from China; mean *C*_*q*_ 32.33). Whilst perhaps not as analytically sensitive as the *Schistosoma* ITS2 real-time PCR when used with *S. mansoni* and *S. haematobium* gDNA, the *S. japonicum* ND1 real-time PCR demonstrated a similar degree of analytical sensitivity to another real-time PCR assay designed to target a 16S mitochondrial DNA locus of *S. mansoni*, specifically [19]. As this assay has been successfully used to detect and diagnose low-intensity human *S. mansoni* infections, these analytical data suggest that the *S. japonicum* ND1 real-time PCR may too be capable of successfully detecting and diagnosing low-intensity human *S. japonicum* infections. This, however, requires clinical validation using human faecal material provided by individuals harbouring low-intensity *S. japonicum* infections*.*

The *S. japonicum* ND1 real-time PCR used here has previously demonstrated a high degree of species specificity [23–25], and it also demonstrated a high degree of species specificity here, both in silico and when challenged using *S. mansoni* and *S. haematobium* gDNA. In addition, the *S. japonicum* ND1 real-time PCR appeared highly specific when challenged using gDNA isolated from naïve human faecal material. This is of particular importance given the great range of non-target DNA present in human faecal material, such as human-, bacterial-, non-human animal-, and plant-derived DNA. In addition to limiting false-positive diagnostic outcomes, false-negative outcomes should also be limited, where possible. When performing PCR-based diagnosis, this is often done by using an internal DNA extraction and PCR internal positive control target locus, such as the PhHV-1 target locus used here. Performing the *S. japonicum* ND1 real-time PCR as a duplex assay that incorporated this additional internal control locus consistently performed well; the PhHV-1 locus was uniformly amplified in all reactions that used template DNA that had been spiked with PhHV-1, confirming successful DNA isolation and PCR reaction steps in all instances whilst having no observable effect on the amplification of the *S. japonicum* ND1 target locus. Again, internal DNA extraction and PCR internal positive control targets are particularly important when using faecal samples, given the many challenges associated with isolating DNA from faecal material [26, 39] as well as the high number of PCR inhibitors present in these samples (such as urea, haemoglobin, heparin, polysaccharides, or chlorophyll originating from consumed vegetables [40, 41]). Whilst other internal DNA extraction and PCR internal positive control targets have been used when diagnosing schistosomiasis using faecal samples, such as the human *β-*actin gene [42], the amount of human DNA in clinical samples cannot be kept consistent across samples, so the addition of a fixed concentration of internal control DNA is generally considered more advantageous than any human-derived internal control DNA target. We therefore recommended that any future studies using the *S. japonicum* ND1 real-time PCR to diagnose *S. japonicum* infections incorporate this additional internal DNA extraction and PCR internal positive control target locus to limit false-negative diagnostic outcomes.

Importantly, the optimised duplex *S. japonicum* ND1 real-time PCR assay was able to consistently produce a *S. japonicum* ND1 amplification curve when used with gDNA isolated from a single *S. japonicum* ovum (mean *C*_*q*_ 31.23), as well as when used with gDNA isolated from 0.2 g naïve filtered faecal material spiked with a single *S. japonicum* ovum (mean *C*_*q*_ 31.67), as found previously when used as a singleplex assay [22]. Whilst no *S. japonicum* ND1 real-time PCR amplification curve was produced when using one of the *Sj-*1egg-stool samples, given the strong performance of the *S japonicum* ND1 real-time PCR when used with the remaining four *Sj-*1egg-stool samples, we posit that this negative outcome in this single sample was likely due to error during the egg-spiking step. As such, our data suggest that the optimised *S. japonicum* ND1 real-time PCR assay used here can in theory reliably detect *S. japonicum* infections that are so low in intensity that the infected individual is shedding ≥ five eggs per gram of faeces (as 0.2 g of faeces is typically used for real-time PCR diagnosis). Again, however, this requires clinical validation using human faecal material from individuals harbouring low-intensity *S. japonicum* infections. Additionally, it should again be noted that our analysis used *S. japonicum* ova derived from China. Whilst in silico analysis suggests that the *S. japonicum* ND1 real-time PCR should perform just as well when using *S. japonicum* isolates from the Philippines, and possibly elsewhere, this should also be confirmed in silico and in vitro using *S. japonicum* parasite material obtained from areas outside of China. Nevertheless, we believe that the optimised *S. japonicum* ND1 real-time PCR assay described here has the potential to become a standard and routinely used method to diagnose low-intensity *S. japonicum* infections and so could be used to support future *S. japonicum* transmission surveillance and elimination programmes, as well as serve as a reliable reference standard when developing and assessing the performance of novel diagnostic assays also designed to diagnose *S. japonicum* infection.

### Study limitations and future work

The diagnostic performance of the optimised *S. japonicum* ND1 real-time PCR assay (with the additional PhHV-1 internal control DNA target) should now be clinically assessed using faecal samples provided by human study participants, or cattle, located in *S. japonicum*-endemic areas, as well *S. japonicum* parasite material obtained from areas outside of China, such as the Philippines. When doing so, comparisons should be made between *S. japonicum* ND1 real-time PCR diagnostic outcomes and other *S. japonicum* diagnostic methods, particularly Kato-Katz faecal egg microscopy. Whilst Kato-Katz faecal egg microscopy can have poor sensitivity when assessing individuals harbouring low-intensity infections, taking multiple faecal samples and preparing multiple Kato-Katz microscopy preparations from the same individual(s) should allow comparisons to be made between, for example, faecal egg count data and the clinical sensitivity of the *S. japonicum* ND1 real-time PCR assay.

Furthermore, although real-time PCR can be highly sensitive and specific, in practice it is used only rarely in schistosomiasis control programmes due to its high cost and need for sophisticated laboratory infrastructure [18]. Further work should be carried out to develop, validate, and standardise highly sensitive and specific DNA-based diagnostic assays that can be carried out at the point of care in resource-poor schistosomiasis-endemic settings, as recommended by WHO [16]. For example, portable and isothermal DNA amplification tools, such as loop-mediated isothermal amplification (LAMP), [43, 44] and recombinase polymerase/aided amplification (RPA/RAA) [45–47], may offer the ability to rapidly and reliably diagnose low-intensity *S. japonicum* infections in endemic settings, without the need for sophisticated laboratory infrastructure. Currently, however, these assays require further development and optimisation before their routine use in disease-endemic areas.

## Conclusions

Here, we demonstrate that the *Schistosoma* ITS2 and the *S. japonicum Sj*TR1 real-time PCR assays are likely incapable of reliably diagnosing low-intensity *S. japonicum* infections. In addition, we demonstrate that the *S. japonicum* ND1 real-time PCR assay can be used with an internal DNA extraction and PCR internal positive control target and may be capable of reliably diagnosing low-intensity *S. japonicum* infections. As this requires further analyses for clarification, clinical assessment of this optimised *S. japonicum* ND1 real-time PCR assay, using faecal samples provided by human study participants or cattle in *S. japonicum*-endemic areas, is encouraged. It is our hope that the optimised *S. japonicum* ND1 real-time PCR assay described here will become a standard and routinely used method to diagnose low-intensity *S. japonicum* infections and will be used to support future *S. japonicum* transmission surveillance and elimination programmes, as well as serve as a reliable reference standard when developing and assessing the performance of novel diagnostic assays also designed to diagnose *S. japonicum* infection.

## Supplementary Information

**Additional file 1: Table S1.** Reference sequences downloaded from the National Center for Biotechnology Information (NCBI; [27] and WormBase ParaSite [28] repositories for in silico specificity testing. **Fig. S1.** Multiple sequence comparison by log-expectation (MUSCLE) alignment of the 77-bp *Schistosoma* ribosomal DNA (rDNA) ITS2 real-time PCR target locus. **Fig. S2.** Multiple sequence comparison by log-expectation (MUSCLE) alignment of the 80-bp *Schistosoma japonicum* TR1 real-time PCR target locus. **Fig. S3.** Multiple sequence comparison by log-expectation (MUSCLE) alignment of the 75-bp *Schistosoma japonicum* ND1 mitochondrial DNA real-time PCR target locus.

**Additional file 2: Text S1.** DNA extraction from adult-stage *Schistosoma* using QIAGEN DNEasy tissue extraction kit.

**Additional file 3: Text S1.** Species confirmation of *Sj*, *Sm*, and *Sh* gDNA isolates. **Table S1.** Primer oligonucleotide sequences used to amplify a 956-bp region of the *Schistosoma* spp. mitochondrial cytochrome oxidase subunit 1 (*cox1*) gene [48]. **Text S2.** Temperature gradient endpoint PCR and Sanger sequencing to confirm real-time PCR target DNA loci and determine optimal real-time PCR annealing temperatures. **Table S2:** Primer oligonucleotide sequences used to amplify a 77-bp fragment of the genus-specific *Schistosoma* spp. internal transcribed spacer 2 (ITS2) ribosomal DNA (rDNA) region [17]. **Table S3:** Primer oligonucleotide sequences used to amplify a *Schistosoma japonicum* 80-bp tandemly repeated region [22]. **Table S4:** Primer oligonucleotide sequences used to amplify a 75-bp fragment of the *Schistosoma japonicum* mitochondrial ND1 gene [25]. **Text S3:** Assessment of real-time PCR primer/probe concentrations and annealing temperatures. **Table S5.**. *Schistosoma* ITS2 real-time PCR primer/probe oligonucleotide concentration combinations tested. **Table S6.** *Schistosoma japonicum* SjTR1 real-time PCR primer/probe oligonucleotide concentration combinations tested. **Table S7.** *Schistosoma japonicum* ND1 real-time PCR primer/probe oligonucleotide concentration combinations tested.

**Additional file 4: Text S1.** DNA extraction from *Schistosoma* spp. ova using QIAGEN DNEasy tissue extraction kit with bead-beat.

**Additional file 5: Text S1.** DNA extraction from 0.2 g faeces using QIAGEN DNEasy tissue extraction kit and MPbio FastPrep bead-beat method.

**Additional file 6: Dataset S1.** *Schistosoma japonicum* 75-bp mitochondrial ND5 target locus 5′–3′.

**Additional file 7: Fig. S1. A.** *Schistosoma japonicum* ND1 real-time PCR analytical sensitivity testing (serially diluted *S. japonicum* gDNA). **B**. The real-time PCR standard curve gave a slope of −3.3493, a *y*-intercept of 42.73, an R^2^ value of 0.997, and a calculated amplification efficiency of 93.3%. **Fig. S2.** *Schistosoma japonicum* ND1 real-time PCR analytical sensitivity testing (H_2_O and naïve faecal material spiked with *S. japonicum* ova and PhHV-1).

## Data Availability

Data supporting the main conclusions of this study are included in the manuscript.

## Abbreviations

BLASTn: Nucleotide Basic Local Alignment Search Tool
bp: Base pair
BRI: Biomedical Research Institute, Maryland, USA
*Cq*: Quantification cycle
DNA/gDNA/rDNA: Deoxyribose nucleic acid/genomic deoxyribose nucleic acid/ribosomal deoxyribose nucleic acid
EVAg: European Virus Archive global
fg: Femtogram
FGS/MGS: Female genital schistosomiasis/male genital schistosomiasis
g: Gram
HL: HyperLadder 100 base pairs (BioLine, UK)
H_2_O: Composition of water (*Hydrogen*_*2*_ + *Oxygen*)
ITS2: Internal transcribed spacer 2
LAMP: Loop-medicated isothermal amplification
MDA: Mass drug administration
NCBI: National Centre for Biotechnology Information
ND1: Mitochondrial NADH dehydrogenase I
ng: Nanogram
NHM: Natural History Museum, London, UK
NTC: No-template negative control
NTD: Neglected tropical disease
PCR: Polymerase chain reaction
PBS: Phosphate-buffered saline
PCR/qPCR: Polymerase chain reaction/quantitative polymerase chain reaction
pg: Picogram
PhHV-1: Phocid herpes virus-1
POC-CCA: Point-of-care circulating cathodic antigen lateral flow assay
PVPP: Polyvinylpolypyrrolidone
RPA: Recombinase polymerase amplification
SCAN: Schistosomiasis Collections at the Natural History Museum, London, UK
*Sj/Sm/Sh*: *Schistosoma japonicum/Schistosoma mansoni/Schistosoma haematobium*
*Sj*TR1: *Schistosoma japonicum* Tandem repeat 1
SSR: Schistosome and Snail Resource, London, UK
SSU: Small subunit
UCP-LF-CAA: Up-converting reporter particle lateral flow circulating anodic antigen assay
UTI: Urinary tract infection
WHO: World Health Organization, Geneva, Switzerland
± 95% CI: ± 95% Confidence intervals
