# Supplementary material for: Optimisation and analytical assessment of a TaqMan™ probe-based real-time PCR assay designed to diagnose infection with Schistosoma japonicum
Source: Parasit Vectors. 2026 Jun 29;19:308. doi: 10.1186/s13071-026-07458-2 (PMC13419353; doi:10.1186/s13071-026-07458-2)
Supplement: Supplementary file 1 — Additional file 1: Table S1. Reference sequences downloaded from the National Center for Biotechnology Information (NCBI; [27] and WormBase ParaSite [28] repositories for in silico specificity testing. Fig. S1. Multiple sequence comparison by log-expectation (MUSCLE) alignment of the 77-bp Schistosoma ribosomal DNA (rDNA) ITS2 real-time PCR target locus. Fig. S2. Multiple sequence comparison by log-expectation (MUSCLE) alignment of the 80-bp Schistosoma japonicum TR1 real-time PCR target locus. Fig. S3. Multiple sequence comparison by log-expectation (MUSCLE) alignment of the 75-bp Schistosoma japonicum ND1 mitochondrial DNA real-time PCR target locus. [file 13071_2026_7458_MOESM1_ESM.pdf]

Optimisation and analytical assessment of a TaqMan™ probe-based real-time PCR assay designed to diagnose infection with *Schistosoma japonicum*

Additional file 1.

Table S1. Reference sequences downloaded from the National Center for Biotechnology Information (NCBI; [1] and WormBase ParaSite [2] repositories for *in silico* specificity testing.

Assay	Reference sequence source	Sequence	Accession number	Strain/isolate	Origin	ID
***Schistosoma* spp. ITS2 real-time PCR**	NCBI [1]	ITS1/ 5.8S/ ITS2	PP963804.1	32B-K	Nigeria	***Sh****-rDNA-1---NIG**
			MK358858.1	Wildtype	Malawi	***Sh****-rDNA-2---MAL**
			GU257398.1	Wildtype	Tanzania	***Sh****-rDNA-3---TAN**
			PQ197034.1	*Sm*-ITSs-H3	Senegal	***Sm****-rDNA-1---SEN**
			KX011042.1	Wildtype	Gabon	***Sm****-rDNA-2---GAB**
			JQ289759.1	ITSd3	Kenya	***Sm****-rDNA-3---KEN**
			FJ859874.1	ZjF6	China	***Sj****-rDNA-1---CHI**
			FJ852573.1	HByx1		***Sj****-rDNA-2---CHI**
			FJ859871.1	YeIIM11		***Sj****-rDNA-3---CHI**
		ITS2	PV282422.1	PJ065	Philippines	***Sh****-rDNA-4---PHI**
		ITS2	PV282421.1	PJ046		***Sh****-rDNA-5---PHI**
		5.8S/ITS2/28S	U22167.1	Sorsogon		***Sh****-rDNA-6---PHI**
***S. japonicum Sj*TR1 real-time PCR**	WormBase ParaSite [2]	Whole genome	PRJNA724792.WBPS19	F4M4	China	***Sj****TR1-1---CHI**
			PRJEA34885.WBPS19	Anhui		***Sj****TR1-2---CHI**
			PRJNA520774.WBPS19	HuSjv2		***Sj****TR1-3---CHI**
	NCBI [1]		025215515.1-ASM2521551v1	F4M4		***Sj****TR1-4---CHI**
			021461655.1-ASM2146165v1	SjaV3_Hu		***Sj****TR1-5---CHI**
			006368765.1-ASM636876v1	HuSjv2		***Sj****TR1-6---CHI**
			000151775.1-ASM15177v1	Anhui		***Sj****TR1-7---CHI**
		*Sj*TR1 target	MW631938.1	Anhui		***Sj*TR1-halili---CHI**
***S. japonicum* ND1 real-time PCR**	NCBI [1]	Whole mitogenome	NC_008074.1	NHM:3375	Mali	***Sh****-mt_genome-1---MALI**
			DQ157222.2	NHM:3375		***Sh****-mt_genome-2---MALI**
			MW067227.1	NHM:3375		***Sh****-mt_genome-3---MALI**

			NC_002545.1	NA	West Africa	***Sm*-mt-genome-1---WA***
		Mitochondrial ND1 gene	AY896693.1	W6000x05	Kenya	***Sm*-mt-ND1-2---KEN***
			AY896670.1	w158m	Liberia	***Sm*-mt-ND1-3---LIB***
		Whole mitogenome	NC_002544.1	Anhui	China	***Sj*-mt-genome-1---CHI***
			HM120841.1	SJZJM6		***Sj*-mt-genome-2---CHI***
			ON637111.1	AHHX01		***Sj*-mt-genome-3---CHI***
			JQ781214.1	SJSORM5	Philippines	***Sj*-mt-genome-4---PHI***
			JQ781215.1	SJYYM2		***Sj*-mt-genome-5---PHI***
			JQ781206.1	SJASNF5		***Sj*-mt-genome-6---PHI***

Where: NCBI: National Center for Biotechnology information [1]; *Sh*: *Schistosoma haematobium; Sm*: *Schistosoma mansoni; Sj*: *Schistosoma japonicum*.

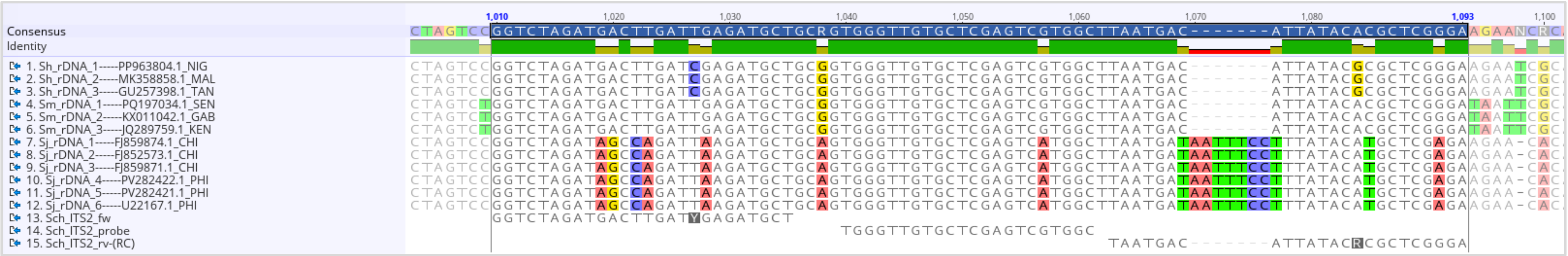

Fig. S1. Multiple sequence comparison by log-expectation (MUSCLE) alignment of the 77 bp *Schistosoma* ribosomal DNA (rDNA) ITS2 real-time PCR target locus. The multiple alignment is comprised of three African *Schistosoma haematobium* ITS1/5.8S/ITS2 sequences, three African *Schistosoma mansoni* ITS1/5.8S/ITS2 sequences, three Asian *Schistosoma japonicum* ITS1/5.8S/ITS2 sequences derived from China, two Asian *S. japonicum* ITS2 sequences derived from the Philippines, and one Asian *S. japonicum* 5.8S/ITS2/28S sequence derived from the Philippines (see Table S1). The *Schistosoma* ITS2 real-time PCR forward and reverse primers, as well as the probe were also included within the alignment. Notably, five single nucleotide polymorphisms (SNPs) were identified in the forward primer region between African and Asian schistosome species (positions 1019, 1020, 1022, 1023, and1028) and one SNP was identified in the probe region between these species (position 1057). In addition, four SNPs were identified in the reverse primer region between these species (positions 1069, 1077, 1085 and 1091), as well as a 7 bp indel between positions 1070 and 1076). ***Where*:** Sh: *S. haematobium;* Sm: *S. mansoni*; Sj: *S. japonicum*; Sch: *Schistosoma* spp.; NIG: Nigeria; MAL: Malawi; TAN: Tanzania; SEN: Senegal; GAB: Gabon; CHI: China; PHI: Philippines; fw: forward primer; rv-(RC): reverse primer reverse-compliment sequence.

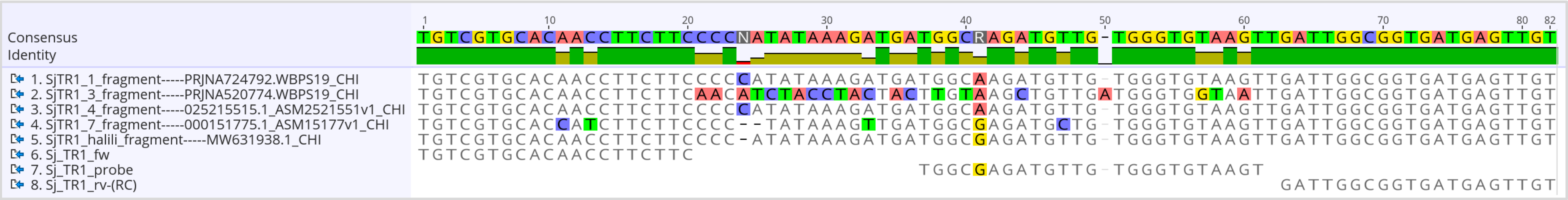

Fig. S2. Multiple sequence comparison by log-expectation (MUSCLE) alignment of the 80 bp *Schistosoma japonicum* TR1 real-time PCR target locus. The multiple alignment is comprised of four target fragments returned from four whole genome sequences derived from China and one *Sj*TR1 locus derived from China [3]. The *S. japonicum Sj*TR1 real-time PCR forward and reverse primers, as well as the probe were also included in within the alignment. Notably, the *Sj*TR1 target fragment was not identified and returned from three of all seven whole genome sequences. Of the four fragments successfully returned from whole genome data, two SNPs were identified in the forward primer region in one of these fragments (aligned sequence number 4; positions 11 and 13) and no SNPs were identified in the reverse primer region. In addition, one SNP was identified in the probe region in three of these four returned fragments (position 41), a single but different SNP was identified in the probe region in the other returned fragment (position 47), and multiple SNPs, as well as an indel, was identified in one of the returned fragments (aligned sequence number two). ***Where*:** Sj: *S. japonicum;* SjTR1: *S. japonicum* tandem repeat 1; CHI: China; fw: forward primer; rv-(RC): reverse primer reverse-compliment sequence.

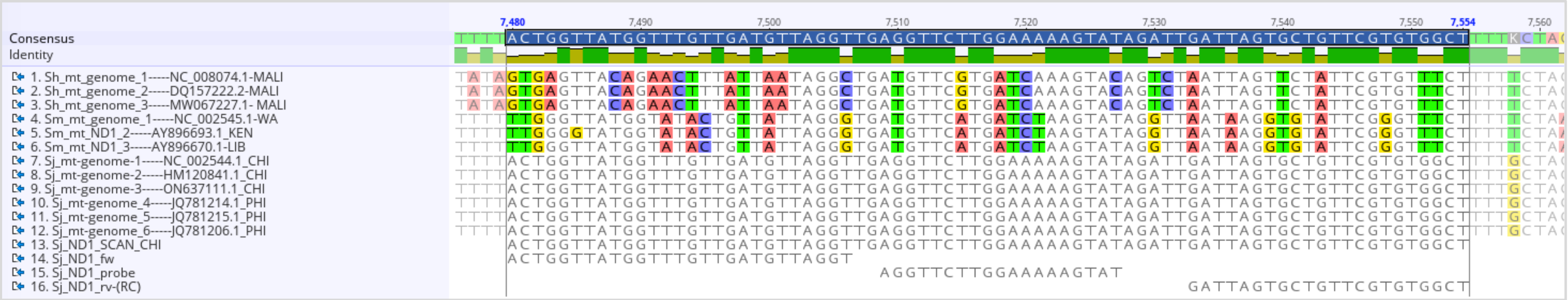

Fig. S3. Multiple sequence comparison by log-expectation (MUSCLE) alignment of the 75 bp *Schistosoma japonicum* ND1 mitochondrial DNA real-time PCR target locus. The multiple alignment is comprised of three African *Schistosoma haematobium* complete mitogenomes, one African *Schistosoma mansoni* complete mitogenome, two African *S. mansoni* mitochondrial ND1 gene sequences, three Asian *Schistosoma japonicum* complete mitogenomes derived from China, three Asian *S. japonicum* complete mitochondrial genomes derived from the Philippines, and one 75 bp *S. japonicum* ND1 consensus sequence generated during here (aligned sequence number 13; strain derived from China). The *S. japonicum* ND1 real-time PCR forward and reverse primers, as well as the probe were also included within the alignment. Eight single nucleotide polymorphisms (SNPs) were identified in the forward primer region between African and

Asian schistosome species (positions 7480, 7481, 7483, 7492, 7494, 7498, 7500 and 7505), with additional SNPs in the forward primer region between *S. haematobium* and Asian schistosome species and *S. mansoni* and Asian schistosome species. A further five SNPs were identified in the probe region between African and Asian schistosome species (positions 7510, 7515, 7518, 7519 and 7520) with additional SNPs in the probe region between *S. haematobium* and Asian schistosome species and *S. mansoni* and Asian schistosome species. In addition, five SNPs were identified in the reverse primer region between African and Asian schistosome species (positions 7533, 7540, 7543, 7551 and 7552) with additional SNPs in the reverse primer region between *S. mansoni* and Asian schistosome species. Notably, no SNPs were identified between any Asian *S. japonicum* species (inclusive of three Chinese and three Philippine sequences), the forward primer, the reverse primer, and the probe across the entire 75 bp target locus. ***Where*:** Sh: *S. haematobium;* Sm: *S. mansoni*; Sj: *S. japonicum*; WA: West Africa; KEN: Kenya; LIB: Liberia; CHI: China; PHI: Philippines; SCAN: Schistosome Collections at the Natural History Museum, London, UK; fw: forward primer; rv-(RC): reverse primer reverse-compliment sequence.

References

1. Geer LY, Marchler-Bauer A, Geer RC, Han L, He J, He S, et al. The NCBI BioSystems database. *Nucleic Acids Res*. 2009;38:492–6. https://doi.org/10.1093/nar/gkp858

2. Howe KL, Bolt BJ, Shafie M, Kersey P, Berriman M. WormBase ParaSite − a comprehensive resource for helminth genomics. *Mol Biochem Parasitol.* 2017;215:2–10. https://doi.org/10.1016/j.molbiopara.2016.11.005

3. Halili S, Grant JR, Pilotte N, Gordon CA, Williams SA. Development of a novel real-time polymerase chain reaction assay for the sensitive detection of *Schistosoma japonicum* in human stool. *PLoS Negl Trop Dis*. 2021;15:e0009877. https://doi.org/10.1371/journal.pntd.0009877
