# Supplementary material for: Optimisation and analytical assessment of a TaqMan™ probe-based real-time PCR assay designed to diagnose infection with Schistosoma japonicum
Source: Parasit Vectors. 2026 Jun 29;19:308. doi: 10.1186/s13071-026-07458-2 (PMC13419353; doi:10.1186/s13071-026-07458-2)
Supplement: Supplementary file 2 — Additional file 2: Text S1. DNA extraction from adult-stage Schistosoma using QIAGEN DNEasy tissue extraction kit. [file 13071_2026_7458_MOESM2_ESM.pdf]

Optimisation and analytical assessment of a TaqMan^{TM} probe-based real-time PCR assay designed to diagnose infection with *Schistosoma japonicum*

Additional file 2.

Text S1: DNA extraction from adult-stage *Schistosoma* using QIAGEN DNEasy tissue extraction kit

Reagents needed (p/1 sample):

180 µl ATL buffer + **20 µl** Proteinase K

200 µl AL buffer

200 µl ethanol

500 µl AW1 solution

500 µl AW2 solution

50/100 µl AE buffer

Protocol

Tissue lysis

1. Add **202 µl** ATL/Proteinase K/PhHV mix to labelled 1.5 mL Eppendorf
2. Add adult *Schistosoma* worm(s)
3. Vortex for **20 seconds**
4. Briefly centrifuge
5. Incubate at **55 ºC** for **2 hours** minimum

DNA extraction

6. Vortex for **20 seconds**
7. Add **200 µl** AL buffer
8. Vortex for **20 seconds**
9. Add **200 µl** ethanol
10. Vortex for **20 seconds**
11. Pipette mixture into spin column
12. Centrifuge for **60 seconds** at **8,000 RPM**
13. Place column in fresh collection tube and add **500 µl** AW1 solution
14. Centrifuge for **60 seconds** at **8,000 RPM**
15. Place column in fresh collection tube and add **500 µl** AW2 solution
16. Centrifuge for **60 seconds** at **8,000 RPM**
17. Re-centrifuge for **2 minutes** at **12,000 RPM**
18. Place column in fresh labelled 1.5 mL Eppendorf
19. Add **50 µl** (if single worm) or **100 µl** (if multiple worms) AE buffer

20. Let stand for 1-2 minutes
21. Centrifuge for **60 seconds** at **8,000 RPM**
22. Again, add **50 µl** (if single worm) or **100 µl** (if multiple worms) AE buffer
23. Let stand for 1-2 minutes
24. Centrifuge for **60 seconds** at **8,000 RPM**
25. Discard spin column; **retaining DNA elution**
26. Short-term storage: 3 – 7 ºC; long-term storage -20 or -80 ºC
