# Supplementary material for: Optimisation and analytical assessment of a TaqMan™ probe-based real-time PCR assay designed to diagnose infection with Schistosoma japonicum
Source: Parasit Vectors. 2026 Jun 29;19:308. doi: 10.1186/s13071-026-07458-2 (PMC13419353; doi:10.1186/s13071-026-07458-2)
Supplement: Supplementary file 3 — Additional file 3: Text S1. Species confirmation of Sj, Sm, and Sh gDNA isolates. Table S1. Primer oligonucleotide sequences used to amplify a 956-bp region of the Schistosoma spp. mitochondrial cytochrome oxidase subunit 1 (cox1) gene [48]. Text S2. Temperature gradient endpoint PCR and Sanger sequencing to confirm real-time PCR target DNA loci and determine optimal real-time PCR annealing temperatures. Table S2: Primer oligonucleotide sequences used to amplify a 77-bp fragment of the genus-specific Schistosoma spp. internal transcribed spacer 2 (ITS2) ribosomal DNA (rDNA) region [17]. Table S3: Primer oligonucleotide sequences used to amplify a Schistosoma japonicum 80-bp tandemly repeated region [22]. Table S4: Primer oligonucleotide sequences used to amplify a 75-bp fragment of the Schistosoma japonicum mitochondrial ND1 gene [25]. Text S3: Assessment of real-time PCR primer/probe concentrations and annealing temperatures. Table S5.. Schistosoma ITS2 real-time PCR primer/probe oligonucleotide concentration combinations tested. Table S6. Schistosoma japonicum SjTR1 real-time PCR primer/probe oligonucleotide concentration combinations tested. Table S7. Schistosoma japonicum ND1 real-time PCR primer/probe oligonucleotide concentration combinations tested. [file 13071_2026_7458_MOESM3_ESM.pdf]

Optimisation and analytical assessment of a TaqMan™ probe-based real-time PCR assay designed to diagnose infection with *Schistosoma japonicum*

Additional file 3.

Text S1: Species confirmation of *Sj, Sm* and *Sh* gDNA isolates

Sj, *Sm* and *Sh* isolates were confirmed as *Schistosoma japonicum, Schistosoma mansoni* and *Schistosoma haematobium*, respectively though end-point PCR and Sanger sequencing of a 956 bp region of the *Schistosoma* spp. mitochondrial cytochrome oxidase subunit I (*cox*1) gene. This was done using previously designed forward (*Schisto-5'*) and reverse (*Schisto-3'*) primers [1], (Table S1). *Cox*1 PCRs were performed using *Sj*, *Sm* and *Sj* template DNA, each in triplicate, and one no-template negative control using nuclease-free H_2O in place of template DNA.

Table S1: Primer oligonucleotide sequences used to amplify a 956 bp region of the *Schistosoma* spp. mitochondrial cytochrome oxidase subunit 1 (*cox*1) gene [1].

Name	Target	Oligonucleotide sequence (5' – 3')	Reference
**Schisto-5'***	Fragment of *Schistosoma* spp. *cox*1 gene.	TCTTTRGATCATAAGCG	[1].
**Schisto-3'†**		TAATGCATMGGAAAAAAACA	

**Forward primer*
†*Reverse primer*

*Cox*1 PCRs were performed using 25 µL PCR reactions made up of 3 µL template DNA that had been normalised to a concentration of 0.3 ng/µL using nuclease-free H_2O, 20 µL nuclease-free H_2O, 400 nM (1 µL of a 10 µmol working solution) of forward and reverse primers [1] and one Illustra PuReTaq ready-to-go PCR bead (Sigma-Aldrich, USA). The following cycling conditions were used: 5 minutes at 95 °C; 40 cycles of 30 seconds at 95 °C, 30 seconds at 40 °C, and 90 seconds at 72 °C; and 10 minutes at 72 °C. Amplicons were visualised and measured by running 4 µL PCR product mixed with 1.5 µL 5x loading buffer blue (Bioline, UK) on a 2% agarose gel stained with GelRed (10 µL GelRed per 100 mL molten agarose) and through use of a 100 bp DNA ladder (New England Biolabs, USA).

All PCR products were purified using the QIAquick PCR purification kit (QIAGEN, UK) according to manufacturer's instructions and Sanger sequenced in the reverse direction using a dilution of the *Schisto-3'* reverse primer. Sequence chromatogram data was visualised and manipulated using Geneious Prime version 2023.01 (Biomatters, LTD). In brief, all *Sj* sequence data were aligned using a multiple alignment with fast fourier transform (MAFFT) alignment (default parameter settings) and trimmed (ensuring uniform ends) to generate a consensus *cox*1 sequence. This consensus sequence was then reverse complimented and analysed using nucleotide Basic Local Alignment Search Tool (BLASTn) analysis within the National Centre

for Biotechnology Information (NCBI) database [2] and was also pairwise aligned to a reference *S. japonicum* whole mitochondrial genome sequence downloaded from the GenBank repository in Geneious Prime, as above [Biomatters, LTD] , (GenBank accession number: NC_002544.1). This was then repeated using all *Sm* sequence data (using GenBank reference *S. mansoni* whole mitochondrial genome sequence: NC_002545.1) and repeated, again using all *Sh* sequence data (using GenBank reference *S. haematobium* whole mitochondrial genome sequence: NC_008074.1).

Text S2: Temperature gradient end-point PCR and Sanger sequencing to confirm real-time PCR target DNA loci and determine optimal real-time PCR annealing temperatures

Schistosoma *ITS2 target locus*

To confirm amplification of the *Schistosoma* ITS2 real-time PCR target DNA locus (a genus-specific 77 bp partial fragment of the tandemly repeated *Schistosoma* spp. ITS2 rDNA region) and to determine optimal real-time PCR annealing temperatures, a temperature gradient end-point PCR was performed using previously designed forward (*Ssp_ITS_48_FW*) and reverse (*Ssp_ITS_124_RV*) primers (Table S2).

Table S2: Primer oligonucleotide sequences used to amplify a 77 bp fragment of the genus-specific *Schistosoma* spp. internal transcribed spacer 2 (ITS2) ribosomal DNA (rDNA) region [3].

**Name**	**Target**	**Oligonucleotide sequence (5' – 3')**	**Reference**
**Ssp_ITS_48_FW** *	*Schistosoma* spp. ITS2	GGTCTAGATGACTTGATYGAGATGCT	[3].
**Ssp_ITS_124_RV** †		TCCCGAGCGYGTATAATGTCATTA	

**Forward primer*
†Reverse primer

The temperature gradient PCR was performed using 25 µL PCR reactions made up of 4 µL *Sj* template DNA that had been normalised to a concentration of 0.25 ng/µL using nuclease-free H_2O, 100 nM of *Schistosoma* ITS2 forward and reverse primers (0.25 µL of a 10 µmol working solution), 8 µL nuclease-free H_2O, and 12.5 µL Luna Universal Probe qPCR Master mix (New England Biolabs (NEB), USA). The following cycling conditions were used: 10 minutes at 95 °C; 40 cycles of 15 seconds at 95 °C, 60 seconds at either 56, 57, 58, 59, or 60 °C, and 60 seconds at 72 °C; and 10 minutes at 72 °C. Each annealing temperature was performed in triplicate and one no-template negative control using nuclease-free H_2O in place of template DNA was used at each annealing temperature. Amplicons were visualised and measured as described above.

All PCR products were purified as described above and Sanger sequenced in both forward and reverse directions using dilutions of the forward (*Ssp_ITS_48_FW*) and reverse (*Ssp_ITS_124_RV*) primers. All sequence chromatogram data was visualised and manipulated as described above. In brief, all forward sequence data were aligned using a multiple alignment with fast fourier transform (MAFFT) alignment (default parameter settings) and trimmed (ensuring uniform ends) to generate a consensus *S. japonicum* ITS2

target locus forward sequence. This was then repeated using all reverse sequence data, and the reverse consensus *S. japonicum* ITS2 target locus sequence was itself reverse complimented. Both consensus sequences were then pairwise aligned using a Needleman-Wunsch alignment (default parameter settings) to generate a complete consensus 77 bp *S. japonicum* ITS2 target locus sequence. This consensus sequence was then analysed using nucleotide Basic Local Alignment Search Tool (BLASTn) analysis within the National Centre for Biotechnology Information (NCBI) database [2] and was also pairwise aligned to a reference *S. japonicum* 18S/ITS1/5.8S/ITS2/28S rDNA reference sequence (1533 bp) downloaded from the GenBank repository in Geneious Prime, as above (GenBank accession number: FJ852573.1). The above protocol was then repeated, however one Illustra PuReTaq ready-to-go PCR bead (Sigma-Aldrich, USA) and 12.5 µL nuclease-free H_2O was used in place of the Luna Universal Probe qPCR Master mix (New England Biolabs (NEB), USA) as this approach is routinely used to PCR amplify and Sanger sequence other *Schistosoma* species DNA loci [4].

Schistosoma japonicum Sj*TR1 target locus*

To confirm amplification of the *S. japonicum Sj*TR1 real-time PCR target DNA locus (a species-specific 80 bp tandemly repeated region) and to determine optimal real-time PCR annealing temperatures, a temperature gradient end-point PCR was performed using previously designed forward (Sj*TR1_FW*) and reverse (Sj*TR1_RV*) primers [5], (Table S3).

Table S3: Primer oligonucleotide sequences used to amplify a *S. japonicum* 80 bp tandemly repeated region [5].

Name	Target	Oligonucleotide sequence (5' – 3')	Reference
***Sj*TR1_FW** *	*S. japonicum Sj*TR1 locus	TGTCGTGCACAACCTTCTTC	[5].
***Sj*TR1_RV** †		ACAACTCATCACCGCCAATC	

**Forward primer*
†*Reverse primer*

The temperature gradient PCR was performed using 25 µL PCR reactions made up of 4 µL *Sj* template DNA that had been normalised to a concentration of 0.25 ng/µL using nuclease-free H_2O, 62.5 nM (0.16 µL of a 10 µmol working solution) forward primer, 500 nM (1.25 µL of a 10 µmol working solution) reverse primer, 7.09 nuclease-free H_2O, and 12.5 µL Luna Universal Probe qPCR Master mix (New England Biolabs (NEB), USA). The following cycling conditions were used: 10 minutes at 95 °C; 40 cycles of 15 seconds at 95 °C, 60 seconds at either 54, 55, 56, 57, 58, 59, 60, 61, or 62 °C, and 60 seconds at 72 °C; and 10 minutes at 72 °C. Each annealing temperature was performed in triplicate and one no-template negative control using nuclease-free H_2O in place of template DNA was used at each annealing temperature. Amplicons were visualised and measured as described above. The above protocol was then repeated, however

one Illustra PuReTaq ready-to-go PCR bead (Sigma-Aldrich, USA) and 12.5 µL nuclease-free H_2O was used in place of the Luna Universal Probe qPCR Master mix (New England Biolabs (NEB), USA).

Schistosoma japonicum *ND1 target locus*

To confirm amplification of the *S. japonicum* ND1 real-time PCR target DNA locus (a species-specific 75 bp fragment of the *S. japonicum* mitochondrial NADH dehydrogenase I (ND1) gene) and to determine optimal real-time PCR annealing temperatures, a temperature gradient end-point PCR was performed using previously designed forward (*Sj_ND1_FW*) and reverse (*Sj_ND1_RV*) primers [6], (Table S4).

Table S4: Primer oligonucleotide sequences used to amplify a 75 bp fragment of the *S. japonicum* mitochondrial ND1 gene [6].

Name	Target	Oligonucleotide sequence (5' – 3')	Reference
**Sj_ND1_FW** *	*S. japonicum* mitochondrial ND1 locus	ACTGGTTATGGTTTGTTGATGTTAGGT	[6].
**Sj_ND1_RV** †		AGCCACACGAACAGCACTAATC	

**Forward primer*
†Reverse primer

The temperature gradient PCR was performed using 25 µL PCR reactions made up of 4 µL *Sj* template DNA that had been normalised to a concentration of 0.25 ng/µL using nuclease-free H_2O, 300 nM (0.75 µL of a 10 µmol working solution) of forward and reverse primers, 7 µL nuclease-free H_2O, and 12.5 µL Luna Universal Probe qPCR Master mix (New England Biolabs (NEB), USA), [6]. The following cycling conditions were used: 10 minutes at 95 °C; 40 cycles of 15 seconds at 95 °C, 60 seconds at either 56, 57, 58, 59, or 60 °C, and 60 seconds at 72 °C; and 10 minutes at 72 °C. Each annealing temperature was performed in triplicate and one no-template negative control using nuclease-free H_2O in place of template DNA was used at each annealing temperature. Amplicons were visualised and measured as described above.

All PCR products were purified as described above and Sanger sequenced in both forward and reverse directions using dilutions of the forward (*Sj_ND1_FW*) and reverse (*Sj_ND1_RV*) primers. All sequence chromatogram data was visualised and manipulated as described above. In brief, all forward sequence data were aligned using a multiple alignment with fast fourier transform (MAFFT) alignment (default parameter settings) and trimmed (ensuring uniform ends) to generate a consensus *S. japonicum* ND1 target locus forward sequence. This was then repeated using all reverse sequence data, and the reverse consensus *S. japonicum* ND1 target locus sequence was itself reverse complimented. Both consensus sequences were then pairwise aligned using a Needleman-Wunsch alignment (default parameter settings) to generate a complete consensus 75 bp *S. japonicum* ND1 target locus sequence. This consensus sequence was then analysed using nucleotide Basic Local Alignment Search Tool (BLASTn) analysis within the National Centre for Biotechnology Information (NCBI) database [2] and was also pairwise aligned to a reference *S. japonicum* whole

mitochondrial genome sequence downloaded from the GenBank repository in Geneious Prime, as above (GenBank accession number: NC_002544.1).

Text S3: Assessment of real-time PCR primer/probe concentrations and annealing temperatures

Schistosoma *ITS2 real-time PCR* for S. japonicum

Table S5. *Schistosoma* ITS2 real-time PCR primer/probe oligonucleotide concentration combinations tested.

	Primer/probe oligonucleotide concentration combinations			
**Real-time PCR oligo**	**1.**	**2[¥].**	**3.**	**4.**
**Ssp_ITS_48_FW***	75 nM	100 nM	125 nM	150 nM
**Ssp_ITS_124_RV[†]**	75 nM	100 nM	125 nM	150 nM
**Ssp_ITS_78T_Pro[‡]**	75 nM	100 nM	125 nM	150 nM

Forward primer
[†]*Reverse primer*
[‡]*Probe*
[¥]*Archer* et al., *2024*

Schistosoma japonicum Sj*TR1 real-time PCR*

Table S6. *Schistosoma japonicum Sj*TR1 real-time PCR primer/probe oligonucleotide concentration combinations tested.

	Primer/probe oligonucleotide concentration combinations				
**Real-time PCR oligo**	**1.**	**2.**	**3.**	**4.**	**5.**
Sj*TR1_FW	62.5 nM[¥]	62.5 nM[¥]	100 nM	125 nM	125 nM
***Sj*TR1_RV[†]**	400 nM	500 nM[¥]	500 nM	500 nM	600 nM
***Sj*TR1_Pro[‡]**	150 nM	200 nM	350 nM	400 nM	500 nM

Forward primer
[†]*Reverse primer*
[‡]*Probe*
[¥]*[6]*

Schistosoma japonicum *ND1 real-time PCR*

Table S7. *Schistosoma japonicum* ND1 real-time PCR primer/probe oligonucleotide concentration combinations tested.

	Primer/probe oligonucleotide concentration combinations			
**Real-time PCR oligo**	**1.**	**2[¥].**	**3.**	**4.**
**Sj_ND1_FW***	150 nM	300 nM	450 nM	600 nM
**Sj_ND1_RV[†]**	150 nM	300 nM	450 nM	600 nM
**Sj_ND1_Pro[‡]**	125 nM	250 nM	375 nM	500 nM

Forward primer

†*Reverse primer*
‡*Probe*
¥*Lier* et al., *2024*

References

1. Lockyer AE, Olson PD, Østergaard P, Rollinson D, Johnston DA, Attwood SW, et al. The phylogeny of the Schistosomatidae based on three genes with emphasis on the interrelationships of *Schistosoma* Weinland, 1858. *Parasitology*. 2003;126:203–24. https://doi.org/10.1017/S0031182002002792

2. Geer LY, Marchler-Bauer A, Geer RC, Han L, He J, He S, et al. The NCBI BioSystems database. *Nucleic Acids Res*. 2009;38:492–6. https://doi.org/10.1093/nar/gkp858

3. Obeng BB, Aryeetey YA, De Dood CJ, Amoah AS, Larbi IA, Deelder AM, et al. Application of a circulating-cathodic-antigen (CCA) strip test and real-time PCR, in comparison with microscopy, for the detection of *Schistosoma haematobium* in urine samples from Ghana. *Ann Trop Med Parasitol*. 2008;102:625–33. https://doi.org/10.1179/136485908X337490

4. Archer J, Cunningham LJ, Juhász A, Jones S, O'Ferrall AM, Rollason S, et al. Molecular epidemiology and population genetics of *Schistosoma mansoni* infecting school-aged children situated along the southern shoreline of Lake Malawi, Malawi. Kumar S, editor. *PLoS Negl Trop Dis.* 2024;18:e0012504. https://doi.org/10.1371/journal.pntd.0012504

5. Halili S, Grant JR, Pilotte N, Gordon CA, Williams SA. Development of a novel real-time polymerase chain reaction assay for the sensitive detection of *Schistosoma japonicum* in human stool. *PLoS Negl Trop Dis*. 2021;15:e0009877. https://doi.org/10.1371/journal.pntd.0009877

6. Lier T, Simonsen GS, Wang T, Lu D, Haukland HH, Vennervald BJ, et al. Real-time polymerase chain reaction for detection of low-intensity *Schistosoma japonicum* infections in China. *Am J Trop Med Hyg.* 2009;81:428–32. https://doi.org/10.4269/ajtmh.2009.81.428
