# Supplementary material for: Optimisation and analytical assessment of a TaqMan™ probe-based real-time PCR assay designed to diagnose infection with Schistosoma japonicum
Source: Parasit Vectors. 2026 Jun 29;19:308. doi: 10.1186/s13071-026-07458-2 (PMC13419353; doi:10.1186/s13071-026-07458-2)
Supplement: Supplementary file 4 — Additional file 4: Text S1. DNA extraction from Schistosoma spp. ova using QIAGEN DNEasy tissue extraction kit with bead-beat. [file 13071_2026_7458_MOESM4_ESM.pdf]

Optimisation and analytical assessment of a TaqMan^{TM} probe-based real-time PCR assay designed to diagnose infection with *Schistosoma japonicum*

Additional file 4.

Text S1: DNA extraction from *Schistosoma* spp. ova using QIAGEN DNEasy tissue extraction kit with bead-beat

Reagents needed (p/1 sample):

2 ml screwcap tube containing **0.45 g** of 1.4 mm ceramic beads
200 µl nuclease-free H_2O
180 µl ATL buffer + **20 µl** Proteinase K **+ 1 µl** PhHV virus (internal control; diluted 1:100)
200 µl AL buffer
200 µl ethanol
500 µl AW1 solution
500 µl AW2 solution
50 µl AE buffer

Protocol

Tissue lysis

1. Decontaminate work area using 10% bleach
2. Add **0.45 g** of 1.4 mm ceramic beads to **2 ml** screwcap tubes containing ova
3. Add **200 µl** nuclease-free H_2O (or volume needed to mimic 200 µl urine sample)
4. Place samples in TissueLyser II and bead-bead for **30 seconds** at **x20 p/second**
5. Add **200 µl** ATL/Proteinase K/PhHV suspension each tube containing miracidia
6. Vortex for **10 seconds**
7. Centrifuge for **30 seconds** at **8,000 RPM**
8. Incubate for **2 hours** or overnight at **56 ºC**

DNA extraction

9. Add **200 µl** AL buffer to each sample
10. Vortex for **10 seconds**
11. Add **200 µl** ethanol to each sample
12. Vortex for **10 seconds**
13. Centrifuge for **30 seconds** at **8,000 RPM**
14. Incubate for **5 minutes** at room temperature
15. Transfer entire lysate to a labelled QIAGEN DNEasy column

16. Centrifuge for **1 minute** at **12,000 RPM**
17. Discard flowthrough
18. Add **500 µl** AW1 solution
19. Centrifuge for **1 minute** at **12,000 RPM**
20. Discard flowthrough
21. Add **500 µl** AW2 solution
22. Centrifuge for **1 minute** at **12,000 RPM**
23. Discard flowthrough
24. Centrifuge for **3 minutes** at **12,000 RPM**
25. Label required number of 1.5 ml Eppendorf tubes
26. Place spin column in corresponding 1.5 ml Eppendorf tube
27. Add **50 µl** AE buffer
28. Incubate for **5 minutes** at **room temperature**
29. Centrifuge for **1 minute** at **12,000 RPM**
30. Discard spin column; **retaining DNA elution**
31. Short-term storage: 3 – 7 ºC; long-term storage -20 or -80 ºC
