# Supplementary material for: Optimisation and analytical assessment of a TaqMan™ probe-based real-time PCR assay designed to diagnose infection with Schistosoma japonicum
Source: Parasit Vectors. 2026 Jun 29;19:308. doi: 10.1186/s13071-026-07458-2 (PMC13419353; doi:10.1186/s13071-026-07458-2)
Supplement: Supplementary file 5 — Additional file 5: Text S1. DNA extraction from 0.2 g faeces using QIAGEN DNEasy tissue extraction kit and MPbio FastPrep bead-beat method. [file 13071_2026_7458_MOESM5_ESM.pdf]

Optimisation and analytical assessment of a TaqMan^{TM} probe-based real-time PCR assay designed to diagnose infection with *Schistosoma japonicum*

Additional file 5.

Text S1: DNA extraction from 0.2 g faeces using QIAGEN DNEasy tissue extraction kit and MPbio FastPrep bead-beat method

Note: Faecal samples should be filtered through a standard 212 µM gauge filter and stored within a labelled 2 mL screwcap tube prior to DNA isolation.

Reagents needed (p/1 sample):

2 ml screwcap tube containing **0.9 g** of 1.4 mm ceramic beads

250 µl of 2% PVPP/PBS suspension

- To make up **100 ml**: 2 g PVPP + 10 ml of 10x PBS + 90 ml ddH_2O… **or** 100 ml 1x PBS + 2 g PVPP)

225 µl ATL buffer + **25 µl** Proteinase K + **2 µl** PhHV-1 virus working stock

- PhHV-1 virus stock diluted 1:100

500 µl AL buffer
400 µl absolute ethanol
500 µl AW1 wash buffer
500 µl AW2 wash buffer
100 µl AE elution buffer

Protocol

Cleaning and rehydrating faecal material stored in ***ethanol*** *(not required if samples are frozen)*

1. Add **0.2 g** faeces to 2 ml screwcap tube containing ceramic beads. Decontaminate utensils using 10% bleach between each faecal sample. If faecal sample is liquified, use P1000 pipette and centrifuge/remove as much H_2O supernatant as possible
2. Include an additional 2 ml screwcap tube containing ceramic beads to act as negative DNA extraction control
3. Add **1.5 ml** H_2O and vortex for **5 seconds**
4. Incubate/agitate at **58 ºC** for **30 minutes**
5. Centrifuge for **1 minute** at **8.000 RPM** and remove as much H_2O supernatant as possible
6. Repeat steps 2, 3 and 4 **twice more** (× 3 washes in total)

Tissue lysis

1. Decontaminate work area using 10% bleach
2. *If spiking faecal material with Schistosoma ova, do this now*
3. Add **250 µl** of 2% PVPP/PBS suspension
4. Vortex for **10 seconds**
5. Centrifuge for **30 seconds** at **8,000 RPM**
6. Freeze sample at **-80 ºC** for **30 minutes – 1 hour** (or -20 ºC overnight)
7. Allow to defrost at room temperature (can take 30 mins – 1 hour; remove tubes from tube rack)
8. Place samples in MPBio FastPrep instrument and beat-bead for **30 seconds** at x**20 p/second**
9. Centrifuge for **30 seconds** at **8,000 RPM**
10. Add **252 µl** ATL/Proteinase K/PhHV-1 suspension
11. Vortex for **10 seconds** (ensure lids are fully sealed)
12. Incubate/agitate **overnight at 55 ºC**

DNA extraction

13. Vortex for **10 seconds**
14. Incubate at **95 ºC** for 10 minutes
15. Centrifuge for **10 seconds** at **8,000 RPM**
16. Add **500 µl** AL buffer
17. Vortex for **30 seconds**
18. Centrifuge for **30 seconds** at **8,000 RPM**
19. Incubate at **70 ºC** for **10 minutes**
20. Label required number of 1.5 ml Eppendorf tubes
21. Add **400 µl** absolute ethanol to each Eppendorf tube
22. Once incubated, vortex samples for **30 seconds**
23. Centrifuge for **30 seconds** at **12,000 RPM**
24. Transfer **800 µl** of supernatant to corresponding Eppendorf containing absolute ethanol
25. Vortex for **30 seconds** (ensure lids are fully sealed)
26. Centrifuge for **10 seconds** at **10,000 RPM**
27. Label required number of spin columns
28. Add **600 µl** sample to corresponding spin column
29. Centrifuge for **60 seconds** at **10,000 RPM**
30. Discard collected waste
31. Add remaining **600 µl** sample to corresponding spin column
32. Centrifuge for **60 seconds** at **10,000 RPM**
33. Discard collected waste

34. Place spin column in fresh 2 ml collection tube
35. Add **500 µl** AW1 solution
36. Centrifuge for **60 seconds** at **10,000 RPM**
37. Discard collected waste
38. Add **500 µl** AW2 solution
39. Centrifuge for **60 seconds** at **10,000 RPM**
40. Centrifuge again for **2 minutes** at **12,000 RPM**
41. Label required number of 1.5 ml Eppendorf tubes
42. Place spin column in corresponding 1.5 ml Eppendorf tube
43. Add **50 µl** AE buffer
44. Incubate at **ambient temperature** for **2 minutes**
45. Centrifuge for **60 seconds** at **8,000 RPM**
46. Return spin column to corresponding 1.5 ml Eppendorf tube
47. Repeat steps 43 - 45
48. Discard spin column; **retaining 100 µl DNA elution**
49. Short-term storage: 3 – 7 ºC; long-term storage -20 or -80 ºC
