# Supplementary material for: Optimisation and analytical assessment of a TaqMan™ probe-based real-time PCR assay designed to diagnose infection with Schistosoma japonicum
Source: Parasit Vectors. 2026 Jun 29;19:308. doi: 10.1186/s13071-026-07458-2 (PMC13419353; doi:10.1186/s13071-026-07458-2)
Supplement: Supplementary file 7 — Additional file 7: Fig. S1. A. Schistosoma japonicum ND1 real-time PCR analytical sensitivity testing (serially diluted S. japonicum gDNA). B. The real-time PCR standard curve gave a slope of −3.3493, a y-intercept of 42.73, an R2 value of 0.997, and a calculated amplification efficiency of 93.3%. Fig. S2. Schistosoma japonicum ND1 real-time PCR analytical sensitivity testing (H2O and naïve faecal material spiked with S. japonicum ova and PhHV-1). [file 13071_2026_7458_MOESM7_ESM.docx]

Optimisation and analytical assessment of a TaqMan^TM^ probe-based real-time PCR assay designed to diagnose infection with *Schistosoma japonicum*

**Additional file 7.**

**Fig. S1.** **A***. Schistosoma japonicum* ND1 real-time PCR analytical sensitivity testing (serially diluted *S. japonicum* gDNA). **B.** The real-time PCR standard curve gave a slope of -3.3493, a *y*-intercept of 42.73, an R^2^ value of 0.997, and a calculated amplification efficiency of 93.3%. ****Where*:** NTC: no-template negative control.

**Fig. S2.** *Schistosoma japonicum* ND1 real-time PCR analytical sensitivity testing (H_2_O and naïve faecal material spiked with *S. japonicum* ova and PhHV-1). PhHV-1 target amplification shown only in ‘naïve_stool’ samples (grey amplification curves) to avoid convoluting figure. ****Where*:** PC: positive control; NTC: no-template negative control.
